# Deciphering early tau pathology events using an Alzheimer’s disease-seeded organotypic hippocampal slice culture model

**DOI:** 10.1016/j.isci.2026.118083

**Published:** 2026-11-02

**Authors:** Rislane Taouili, Camille Lefebvre, Marie Oosterlynck, Thomas Bouillet, Sabiha Eddarkaoui, Dylan Musiol, Coraille Formentin, Bertrand Accart, Claude-Alain Maurage, Morvane Colin, Luc Buée, Sophie Halliez

**Affiliations:** 1 University Lille, Inserm, CHU Lille, U1172 - LilNCog - Lille Neuroscience & Cognition, 59000 Lille, France; 2 University Lille, CHU-Lille, CRB/CIC1403, Centre de Ressources Biologiques du Centre d'Investigation Clinique, Lille, France

**Keywords:** tau seeding, human brain-derived tau seeds, bioassay, tau phosphorylation, pS422, pT217, prion-like, Alzheimer’s disease, tauopathy, organotypic brain slice culture

## Abstract

In Alzheimer’s disease, the spreading of the tau pathology through the brain is assumed to rely on pathological species of tau acting as seeds. To better understand the underlying mechanisms, we need models allowing us to study tau seeding in a brain-like environment. We developed an *ex vivo* model based on organotypic hippocampal slice cultures prepared from transgenic mouse pups expressing human pro-aggregative mutant tau. We show for the first time that human brain-derived tau seeds are able to potentiate tau pathology in such cultures. Moreover, tau seeds are generated *ex vivo*, completing the validation of the model. Then, we shed light on the initial events of tau seeding: tau pathology appears sequentially, first in neurites and then in cell bodies, and misfolding (MC1+) precedes pathological phosphorylation (pS422, pT217). Altogether, this makes the present model a highly valuable bioassay to decipher the molecular and cellular mechanisms leading to tau pathology development.

## Highlights

• AD tau seeds potentiate tau pathology development in OHSC in less than two weeks
• Seeded OHSC constitute a new tool to study tau seeding in a brain-like environment
• Tau pathology appears sequentially, first in neurites and then in cell bodies
• Tau misfolding (MC1+) precedes pathological phosphorylation (pS422 and pT217)

## Graphical abstract

**Figure:**
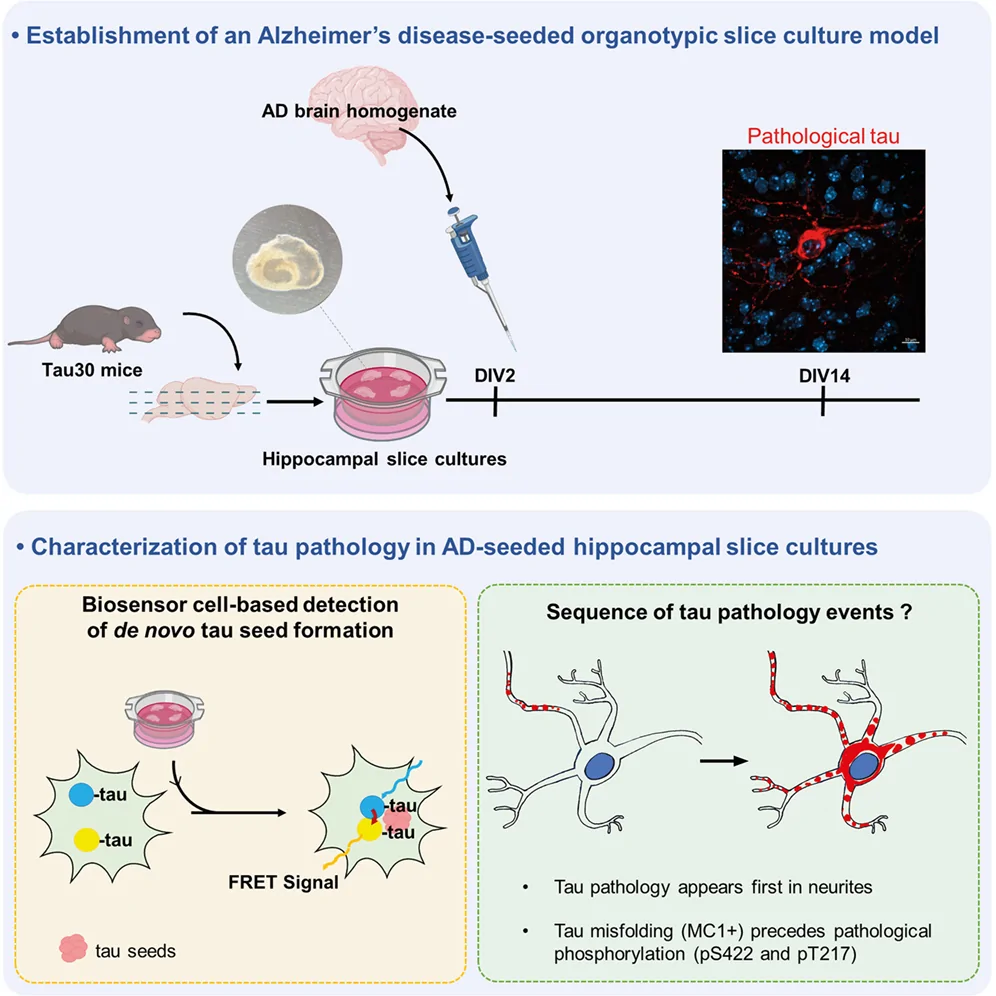

## Teaser

Cellular neuroscience; Molecular neuroscience

## Introduction

Tauopathies are a group of about 25 neurodegenerative diseases, which, although clinically distinct, are all characterized by the progressive loss of neurons and synapses due to abnormal deposition of tau protein into intracellular fibrillary structures. Alzheimer’s disease (AD) is classified as a tauopathy and is the most common neurodegenerative disease.^1^ AD is characterized by the accumulation and aggregation in the brain of amyloid-β peptides (Aβ) and abnormally and hyper-phosphorylated tau proteins that culminate in extracellular amyloid plaques and intracellular neurofibrillary tangles (NFT), respectively.^2^ However, while Aβ pathology starts earlier, tau pathology correlates better with neuronal loss and cognitive decline,^3^ correlating tau misfolding/aggregation with neurodegeneration. In the human brain, there are several tau protein isoforms that result from alternative splicing of the primary RNA transcript of the tau gene *MAPT* (for microtubule-associated protein tau), to which are added numerous post-translational modifications (PTM).^4^ Tau isoforms can be classified into isoforms with a microtubule-binding domain consisting of four repeat sequences collectively referred to as 4R tau, and isoforms with a microtubule-binding domain consisting of three repeat sequences collectively referred to as 3R tau.^5^ The composition of tau aggregates in 3R tau and 4R tau varies depending on the tauopathy, both of which are present in NFT observed in AD. In neurons, tau is mainly known as an axonal and microtubule-associated protein, although it is also located, to a lesser extent, in other neuronal compartments^6^ and exhibits a wide range of functions.^4^ In AD, hyper- and abnormally phosphorylated aggregated tau relocates massively in the somatodendritic compartment.^2^ Currently, it remains unclear whether the different AD-associated tau phosphorylation sites appear before or after tau misfolding and aggregation. Thus, tau phosphorylation could potentially favor/induce these changes or, rather, be a consequence of the misfolding and aggregation.^7^^,^^8^^,^^9^^,^^10^^,^^11^

Some tauopathies, especially AD, exhibit stereotypical pathology staging patterns among neuroanatomically connected brain regions, defined as Braak stages.^2^ This pattern of progression, combined with the demonstration of tau seeding activity notably through *ex cellulo* amplification tests, such as real-time quaking-induced conversion (RT-QuIC)^12^ and protein misfolding cyclic amplification (PMCA)^13^ assays, has led to the idea that the tau pathology can spread through the brain via prion-like mechanisms.^5^^,^^14^ According to the prion-like hypothesis, some pathological tau species act as seeds that have the ability to recruit and transmit their core structure to normal tau and, thus, to spread tau pathology through the brain cells. As a result, tau seeding activity in brain tissues from patients with AD correlates with the aggressiveness of the clinical disease.^15^ However, finely deciphering the cellular mechanisms involved in a disease such as AD that progresses over several decades remains challenging. Most pre-clinical studies rely on mouse models of tauopathy that overexpress human tau with specific mutations involved in tauopathies that increase tau propensity to aggregate. The transgenic THY-tau30 line (or tau30 mice) is one of these models. These mice express the 1N4R human tau isoform bearing two pathogenic mutations (P301S and G272V) under the control of the neuron-specific Thy1.2 promoter, thereby spontaneously developing tau pathology around 3 months, subsequent memory deficits, and finally neuronal loss around 1 year.^16^ As in other models, this further validates the causative link between tau misfolding/aggregation (here, favored by mutations) and neurodegeneration. Importantly, tau pathology development in tau30 mice (or other models) can be potentiated by the intracerebral injection of human brain materials derived from patients with AD,^17^ allowing the ability to assay the tau seeding activity of a given biological sample. While *in vivo* models remain powerful for deciphering molecular and cellular mechanisms, their complexity poses significant challenges. In contrast, cellular models offer greater accessibility and versatility,^18^^,^^19^^,^^20^^,^^21^^,^^22^ including the fluorescence resonance energy transfer (FRET)-based biosensor cell line developed by Marc Diamond’s team.^23^ However, most cellular models fail to recapitulate neuronal features, and brain networks present even greater experimental hurdles.

Organotypic brain slice cultures address this gap by preserving cellular diversity, spatial architecture, and synaptic connectivity, effectively bridging simplified cell culture models and *in vivo* systems.^24^ Recent studies demonstrate that these cultures sustain tau pathology development,^25^^,^^26^^,^^27^^,^^28^^,^^29^^,^^30^ enabling investigation of tau pathology progression in a near-physiological context while retaining the practical advantages of cell culture models. For instance, slice cultures from triple-transgenic 3xTg-AD mouse pups (expressing mutant human amyloid precursor protein (APP), presenilin-1, and tau P301L) accumulate APP, Aβ, and tau, with progressive increases in tau phosphorylation and synaptic accumulation over time.^27^ Viral vector-induced overexpression of double (P301L/S320F) or triple (A152T/P301L/S320F) mutated human tau in organotypic brain slice cultures leads to spontaneous neurofibrillary inclusions within two weeks and neurodegeneration by two months.^29^ Similarly, overexpression of the mutated tau repeat domain (RD) in organotypic hippocampal slice cultures (OHSC) induces neurodegeneration within three weeks.^26^ Moreover, OHSC from P301S tau transgenic pups develop tau pathology through homo-seeding processes, as P301S tau seeds from adult mouse brain homogenates potentiate tau pathology when added to the culture medium.^30^ However, these seeds remain mouse-derived. Developing AD-seeded OHSC would thus provide a valuable *ex vivo* model for studying tau pathology induced by human AD brain-derived tau seeds.

In the present study, we adapted published protocols^30^^,^^31^ to potentiate tau pathology in less than two weeks in OHSC prepared from tau30 pups upon treatment with human brain homogenates (BH) derived from patients with AD. The number of neuronal bodies positive for tau pathology per slice and, to a lesser extent, the quantity of tau seeds produced *ex vivo* correlated with the seeding potential exhibited by the initial BH used to treat the OHSC. We also investigated the sequential features of tau pathology development in this new *ex vivo* model to contribute to the ongoing debate about the initial molecular events in AD. We show that (1) tau pathology appears sequentially first in neurites and then in cell bodies and (2) tau misfolding (recognized by the MC1 antibody) precedes pathological tau phosphorylation (pS422 and pT217) in a context of tau seeding. This makes AD-seeded OHSC a reliable bioassay for tau seeding activity, allowing to decipher the molecular and cellular mechanisms involved in AD and other tauopathies.

## Results

### Characterization of the human brain homogenates derived from patients with AD and non-demented controls

To develop a reliable protocol to generate AD-seeded OHSC, we prepared several BH from frozen brain samples obtained from 5 patients with AD (AD1 to AD5) and 5 non-demented controls (Ctl1 to Ctl5) whom main characteristics are presented in Table 1. All the homogenates were characterized by immunoblots regarding tau content (Figure 1A; Figure S1). This revealed that they harbored different levels of total tau (detected using a tau C-terminal (Cter) antibody), phosphorylated tau at residue serine 422 (detected using the 2H9 antibody), and phosphorylated tau at both residues threonine 212 and serine 214 (detected using the AT100 antibody). Phosphorylated tau was found to be clearly higher in all the AD BH compared to the control BH. Total tau was also found to be higher in the AD BH, except for one (AD3), which also showed the lowest amount of phosphorylated tau among the AD BH (Figure 1A).

**Table 1:** Demographic, biological, and clinical characteristics of the human brain sample donors/brain samples used (*n* = 5 patients with AD, *n* = 5 non-demented controls)

	Patient	Sex	Age	PMD (h)	Braak stage	Thal stages	Region sampled
AD	1	M	57	22	6	–	parietal cortex
	2	M	67	9,5	6	4	parietal cortex
	3	F	86	10	6	–	parietal cortex
	4	M	64	20	6	4	prefrontal cortex
	5	F	60	24	5/6	5	prefrontal cortex
Ctl	1	M	22	24	/	/	parietal cortex
	2	F	81	8	/	/	prefrontal cortex
	3	F	76	14	/	/	parietal cortex
	4	F	78	14	/	/	prefrontal cortex
	5	M	75	7	/	/	parietal cortex

PMD, post-mortem delay.

**Figure 1:**
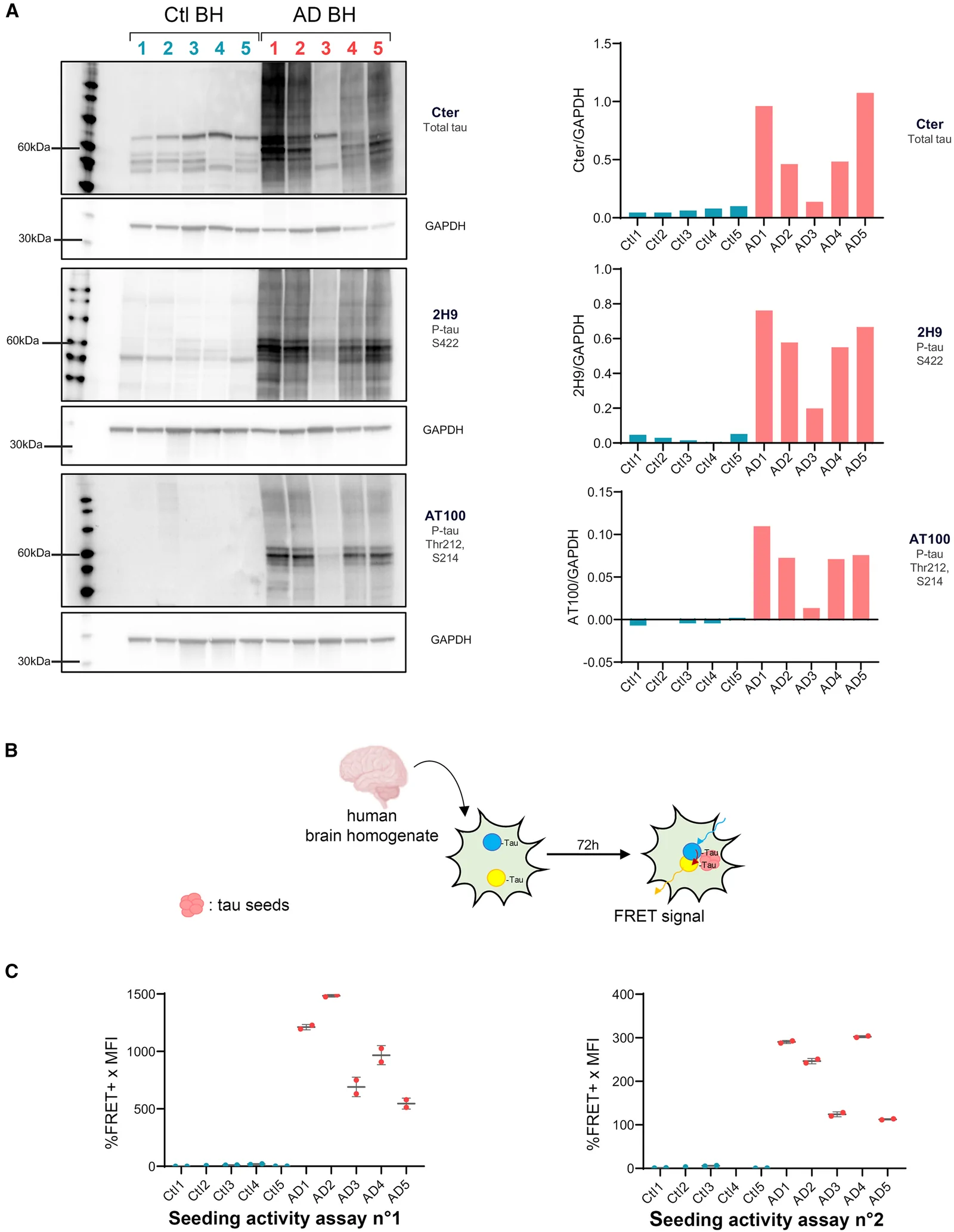
Characterization of the human brain homogenates (A) Biochemical characterization of the human BH obtained from non-demented controls (Ctl, *n* = 5) and patients with AD (AD, *n* = 5) by western blot analysis using anti-tau antibodies detecting total tau (Tau Cter), phosphorylated tau at residue serine 422 (2H9), and phosphorylated tau at both residues threonine 212 and serine 214 (AT100). Glyceraldehyde-3-phosphate dehydrogenase (GAPDH) was used as a loading control. Blots (left) and relative quantifications (right) are presented. (B) Schematic representation of the FRET-based biosensor cell assay. Equal amounts of each BH (40 μg of total protein) were applied to the cells, and the FRET signal was quantified using flow cytometry. (C) Results are expressed as %FRET x MFI (1 dot = 1 replicate). Two independent assays were performed (left and right).

The tau seeding capacity of the human BH was estimated using the FRET-based biosensor cell line.^23^ Briefly, these cells express soluble forms of mutated tau RD coupled to cyan fluorescent protein (CFP) or yellow fluorescent protein (YFP). Upon addition of a seed, both molecules are brought in close proximity, allowing an energy transfer between CFP and YFP that is detectable/quantifiable by flow cytometry (Figure 1B). This only happens when external tau seeds, like the ones contained in the AD BH, are introduced into the cells and initiate a nucleation process, making this assay a very reliable way to estimate tau seeding activity among human BH.^23^ Such an assay was repeated twice and in duplicate for each human BH. As expected, all the BH obtained from the patients with AD showed some tau seeding activity, while no seeding activity was observed for the BH obtained from the control subjects (Figure 1C). However, we observed some differences between the results of the two assays (performed three months apart with different vials of amplified biosensor cells), although three AD BH (AD1, AD2, and AD4) were found consistently with a higher tau seeding activity than the other two (AD3 and AD5).

### Generation of AD-seeded organotypic hippocampal slice cultures

We first used AD1 and Ctl1 (used as a control) to develop a reliable protocol to generate AD-seeded OHSC. OHSC were established from postnatal day (P) 7 tau30 mice and wild-type (WT) littermates (used as controls). Tau30 OHSC were observed to maintain cellular diversity and spatial architecture (Figure S2). After two days *in vitro* (DIV), human BH (80 μg/mL of total protein) obtained from patients with AD or non-demented controls was added overnight into the slice culture medium (SCM). Then, the SCM was replaced with fresh SCM, and the slices were kept in culture for 12 additional days (up to 14 DIV in total) (Figure 2A). Cells harboring pathological tau stained by the MC1 antibody, to detect pathological conformations of tau,^7^^,^^32^ were observed in tau30 OHSC treated with 80 μg/mL AD BH (Figure 2B, left). MC1+ cells were observed neither in tau30 OHSC treated with control BH nor in WT OHSC treated with AD BH (Figure 2B, middle, right). This clearly demonstrates that the MC1+ signal was not due to residue from the applied homogenate itself and that only the application of AD BH can potentiate tau pathology in tau30 OHSC. Control BH-treated tau30 OHSC and maintained tau30 OHSC without treatment of BH never developed tau pathology, even after four weeks of culture (Figures S3 and S4). The amount of human BH added to the SCM (80 μg/mL of total protein) was optimized to lead to a higher number of MC1+ cell bodies per slice for AD1 at 14 DIV, without compromising general cell viability, as assessed by propidium iodide staining (Figures 2C and 2D). Moreover, still using AD1, we evaluated the number of MC1+ cell bodies per slice over time. We observed an increase in the number of MC1+ cell bodies with culture time (Figure S5), which was also observed with AD2 (data not shown). In coherence with the expression pattern of human tau that is mostly restricted to the CA1 hippocampal sub-region in tau30 P7 mice (Figure S6), MC1+ cell bodies were only found located in the CA1 area in AD-seeded OHSC (Figure 2B).

**Figure 2:**
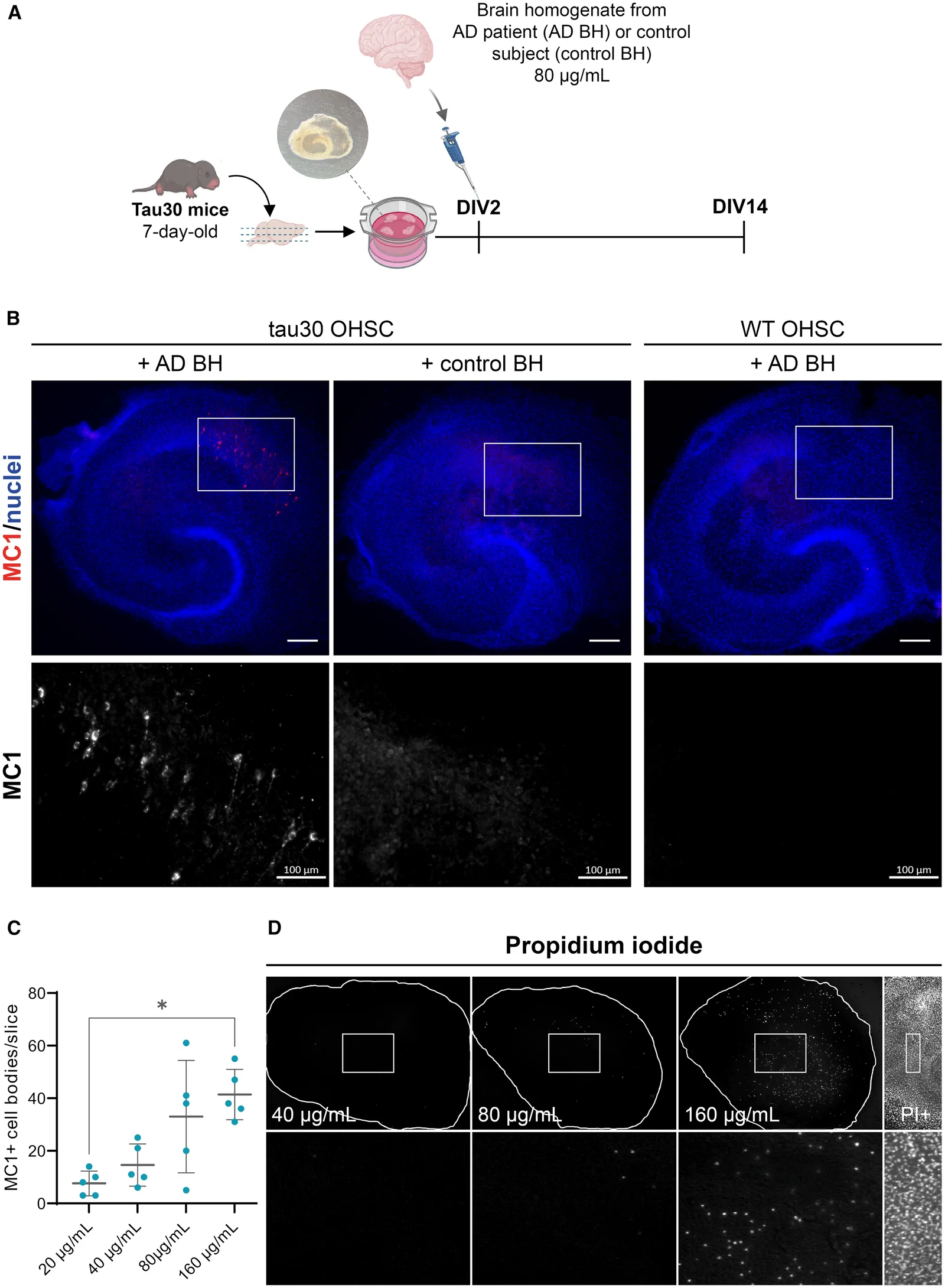
Potentiation of tau pathology in tau30 organotypic hippocampal slice cultures (A) Schematic representation of the experimental protocol developed in this study. Briefly, OHSC are established from P7 tau30 mice. After two DIV, human BH (80 μg/mL of total protein) obtained from patients with AD (or control subjects) is added overnight to the culture medium. Then, the medium is replaced with fresh medium, and the slice cultures are maintained until a total of 14 DIV. (B) Potentiation of tau pathology shown by the presence of MC1 staining (conformational anti-tau antibody, shown in red) in a representative 14-DIV tau30 OHSC treated at 2 DIV with AD BH (AD1; top left). Both 14-DIV tau30 OHSC treated at 2 DIV with control human BH (Ctl1; top middle) and 14-DIV WT OHSC treated at 2 DIV with AD BH (AD1; top right) were negative for MC1 staining. The nuclei were stained with DAPI (in blue). Wide-field acquisitions (top) and CA1-focused acquisitions (bottom) are presented. The acquisitions were performed using a fluorescence microscope and a spinning disk microscope (20× objective), respectively. Scale bars, 200 μm (top) and 100 μm (bottom). (C) Quantification of MC1+ cell bodies in 14-DIV tau30 OHSC exposed to increasing concentrations of AD1 at 2 DIV (*n* = 5 per condition). A significant increase in the number of MC1+ cell bodies was observed with the highest BH protein concentration. ∗: *p* < 0.05, Dunn’s multiple comparisons test. (D) Propidium iodide (PI) assay. Representative images of slice cultures treated with 40, 80, and 160 μg/mL of AD1. Higher magnifications are also presented (bottom). The application of up to 80 μg/mL of BH did not compromise cell viability (*n* = 5 per condition). PI+, positive control.

### The *ex vivo* generated tau lesions within OHSC are associated with the presence of tau seeds

Having established the optimal conditions under which our OHSC model reliably supports tau seeding, we next evaluated the model’s sensitivity and reproducibility. For this, we applied the finalized protocol to the five different AD BH and the five control BH, previously characterized. As a first readout of the *ex vivo* potentiation of tau pathology, we quantified the number of MC1+ cell bodies per slice obtained at 14 DIV. As expected, we never detected MC1+ signal in the tau30 OHSC when treated with any of the control human BH (Figure 3A). Similarly, we also never obtained any MC1+ staining in the WT OHSC when treated with any of the AD BH (data not shown). In contrast, all the tau30 OHSC treated with AD BH showed MC1+ cell bodies (Figure 3A).

**Figure 3:**
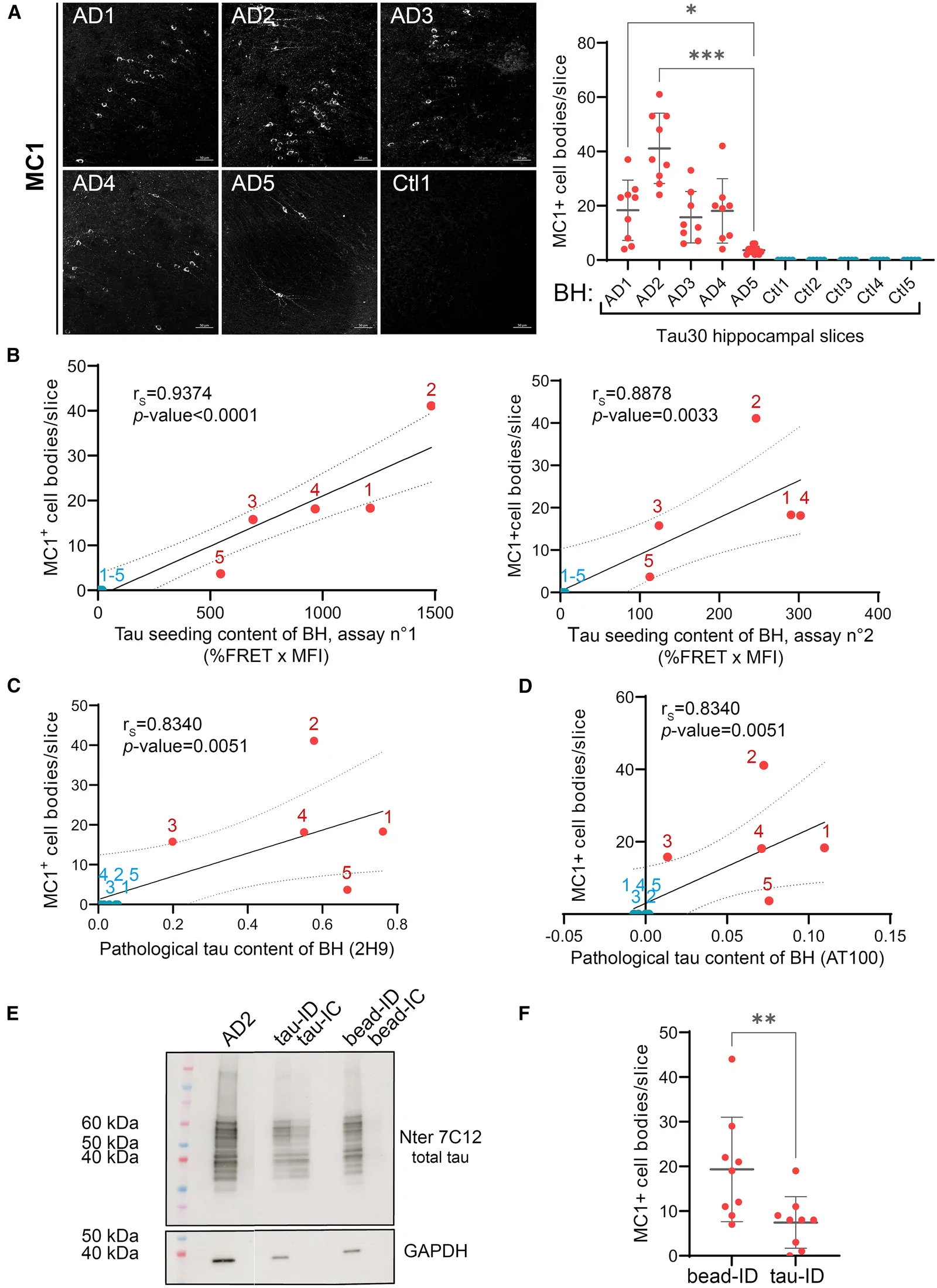
Quantifiable and reproducible potentiation of tau pathology by AD tau seeds in organotypic hippocampal slice cultures (A) Representative images of MC1 labeling in 14-DIV tau30 OHSC treated at 2 DIV with human AD BH (1–5) or control BH (Ctl1) (left). Acquisitions were performed using a spinning disk microscope (20× objective). Scale bars, 50 μm. (Right) Number of MC1+ cell bodies per 14-DIV tau30 OHSC treated with AD BH (AD1–5, *n* = 8–10 for each condition, represented by red circles) or Ctl BH (Ctl1–5, *n* = 5 for each condition, represented by blue circles) at 2 DIV. ∗: *p* < 0.05 and ∗∗∗: *p* < 0.001, Dunn’s multiple comparisons test performed for AD BH. (B) Mean number of MC1+ cell bodies per BH-treated tau30 OHSC obtained for each human BH (AD BH and control BH represented in red and blue, respectively) plotted as a function of the tau seeding content of the BH measured by each of the two FRET-based biosensor cell assays presented in Figure 1C. r_s_ and *p* values are reported on the graphs. The linear regression lines and respective confidence intervals were added under the assumption of a linear relationship. (C and D) Mean number of MC1+ cell bodies per BH-treated tau30 OHSC obtained for each human BH (AD BH and control BH represented in red and blue, respectively) plotted as a function of the pathological tau content of the BH quantified from the immunoblots performed with 2H9 (C) or AT100 (D) and anti-GAPDH antibodies presented in Figure 1A. r_s_ and *p* values are reported on the graphs. The linear regression lines and respective confidence intervals were added under the assumption of a linear relationship. (E) Western blot analysis using anti-tau antibody detecting total tau (Nter 7C12) and anti-GAPDH antibody performed on BH from a patient with AD, same AD BH after tau-immunodepletion (tau-ID) or after incubation with the beads alone (bead-ID), immunocaptured tau fraction (tau-IC), and corresponding negative control (bead-IC). (F) Number of MC1+ cell bodies per 14-DIV tau30 OHSC treated with tau-immunodepleted AD BH (tau-ID) or AD BH pre-incubated with the beads alone (bead-ID) at 2 DIV (*n* = 9 per condition). ∗∗: *p* < 0.01, Mann-Whitney U test.

Moreover, the number of MC1+ cell bodies per slice varied according to the BH applied (Figure 3A). AD5 in particular led to the fewest MC1+ cells per slice, although it displayed average amounts of AT100+ and 2H9+ tau compared to the other AD BH (Figure 1A). However, it was the AD BH with the lowest tau seeding activity (Figure 1C). So, we questioned whether the level of *ex vivo* potentiation was related to the amount of phosphorylated tau (measured by immunoblots, Figure 1A) and/or tau seed level (quantified using the tau FRET-based biosensor cell assay, Figure 1C) contained in each homogenate. This was addressed by calculating a Spearman’s rank correlation coefficient (r_s_) that allows assessment of whether two variables are monotonically related, although not necessarily in a linear relationship. The highest correlation was obtained between the average number of MC1+ cell bodies per slice and the tau seeding content of the BH (*r*_s_ = 0.9374 with *p* < 0.0001 and *r*_s_ = 0.8878 with *p* = 0.0033 for the values obtained with, respectively, the first and the second seeding activity assay using the FRET-based biosensor cell line) (Figure 3B). Further, a strong correlation (*r*_s_ = 0.8340 with *p* = 0.0051) was also observed with the amount of 2H9+ tau (Figure 3C), and with the amount of AT100+ tau (Figure 3D) and, to a lesser extent (*r*_s_ = 0.7693 with *p* < 0.05), with the amount of total tau (Figure S7A). Since the amount of total tau varied greatly between the different human BH, we normalized the amounts of pathological tau to the amount of total tau (Figure S7B). We observed that the level of *ex vivo* potentiation still correlated with the normalized amount of AT100+ tau (*r*_s_ = 0.9245 with *p* = 0.0003) and, to a lesser extent (*r*_s_ = 0.6918 with *p* < 0.05), with the normalized amount of 2H9+ tau (Figure S7C). All of this strongly suggests that the tau pathology potentiation in tau30 OHSC resulted from the seeding activity harbored by some of the pathological tau species contained in the AD BH. To validate this further, we immunodepleted tau from AD BH using magnetic beads coated with anti-tau antibody (tau N-terminal (Nter) 7C12) (Figure 3E; Figure S8). The tau immunodepleted fraction (tau-ID) showed successful loss of tau, appearing in the tau immunocaptured fraction (tau-IC). Potentiation of tau pathology in tau30 OHSC with the resulting tau-immunodepleted AD BH resulted in significantly less MC1+ cell bodies after 14 DIV compared to the control condition where AD BH was incubated beforehand with uncoated magnetic beads (Figure 3F). This validates the instrumental contribution of tau seeds in this *ex vivo* model.

According to the prion-like hypothesis, tau seeding occurring in OHSC should result in the generation of pathological tau conformers (as demonstrated by MC1+ signal), with at least some of them harboring tau seeding activity. To validate the *de novo* generation of tau seeds into slice cultures, we collected and homogenized 14-DIV tau30 OHSC or WT OHSC treated with AD or control BH. The tau seeding content of the resulting OHSC homogenates was estimated using the FRET-based biosensor cell line (Figure 4A). This was performed with two independent series of 14-DIV OHSC. In both experiments, the FRET signal was consistently found higher for all AD BH-treated tau30 OHSC compared to both control BH-treated tau30 OHSC and AD BH-treated WT OHSC (Figure 4B). This clearly demonstrates that the tau seeds found in AD-seeded tau30 OHSC were mostly formed during the two weeks of culture and were not just some residual tau seeds present in the homogenates we initially applied, although we observed intra-assay and inter-assay variations. Lastly, we examined whether the average number of MC1+ cell bodies correlated with the tau seeding content of the OHSC at the end of the culture for a given human BH used to treat the slice cultures. We calculated the r_s_ between MC1+ cell bodies per slice and the tau seeding content (Figure 4B). We observed a strong correlation (*r*_s_ = 0.8728 with *p* = 0.0022 and *r*_s_ = 0.7486 with *p* = 0.0311 for the values obtained with, respectively, the first and the second seeding activity assay). Collectively, these results indicate that the tau30 OHSC, after addition of AD BH, acquires a high seeding capacity, which is correlated with the number of formed tau lesions (MC1+) and thus with the initial seeding capacity of the added BH.

**Figure 4:**
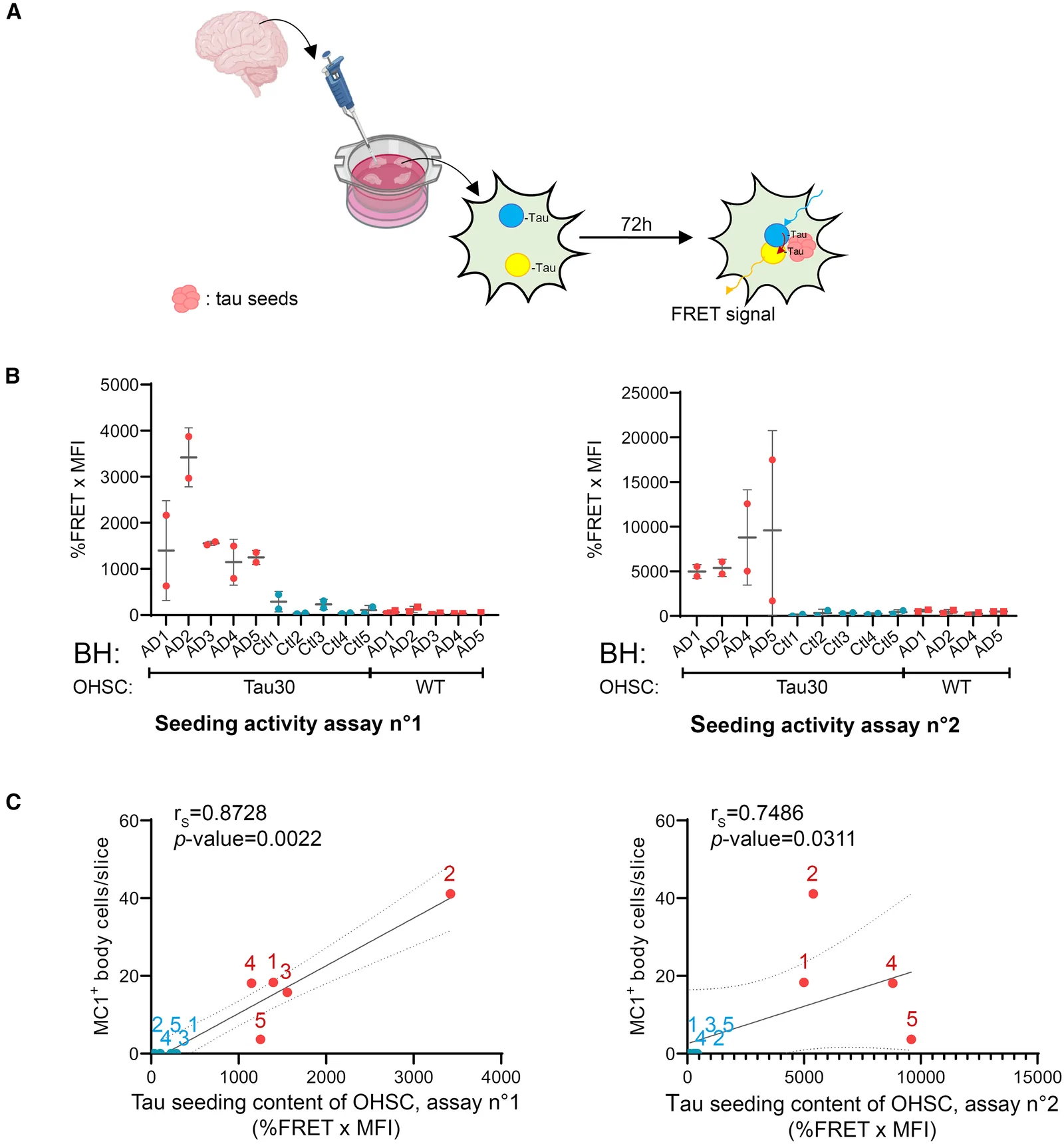
*De novo* production of tau seeds in AD-seeded organotypic hippocampal slice cultures (A and B) Schematic representation of the experimental workflow (A): 14-DIV tau30 and WT OHSC treated at 2 DIV with human BH were harvested, homogenized, and then applied to tau FRET-based biosensor cells in order to estimate their seeding activity content. The FRET signal was quantified using flow cytometry, and the results (B) are expressed as %FRET × MFI (1 dot = 1 replicate). Two independent assays were performed (note that AD3 could not be reanalyzed due to the lack of human sample availability). (C) Mean number of MC1+ cell bodies per BH-treated tau30 OHSC obtained for each human BH (presented in Figure 3A, with AD BH and control BH represented in red and blue, respectively) plotted as a function of the tau seeding content measured in the OHSC via the two assays presented in (B). r_s_ and *p* values are reported on the graphs. The linear regression lines and respective confidence intervals were added under the assumption of a linear relationship.

### The *ex vivo* model allows to decipher the sequential events leading to tau pathology

Taken together, the previous results showed that, in our *ex vivo* model, tau seeding occurred as expected, resulting in the generation of abnormally conformed tau, including tau seeds, making AD-seeded OHSC highly suitable to decipher the steps leading to the development of tau pathology. To start, neuronal cell loss and microglial response were grossly assessed via the quantification of, respectively, NeuN+ nuclei and Iba1+ area in the CA1 subregion around the tau lesions. Neither neuronal loss nor microglial response was observed in the AD-seeded OHSC compared to the controls at 14 DIV (Figure S9). Next, we took advantage of this new model to investigate the temporal relationship between tau misfolding (highlighted by MC1+ staining) and some tau PTM. We selected abnormal and/or pathological tau phosphorylation sites: tau phosphorylation at residue serine 396 (pS396), tau phosphorylation at residues serine 202, threonine 205, and serine 208 (pS202/pT205/pS208), detected with the AT8 antibody, tau phosphorylation at residue serine 422 (pS422), and tau phosphorylation at residue threonine 217 (pT217).

Some data link pS396 with progression of AD and other tauopathies,^33^ while other data show that it can happen in specific, but non *per se* pathological, conditions.^34^^,^^35^ In AD-seeded tau30 OHSC, the MC1+ neuronal cells observed at 14 DIV were all found pS396+, while neighboring cells were only pS396+ (Figure 5A). pS396+ cells (negative for MC1 signal) were also present in the CA1 area of tau30 OHSC treated with control BH, and to a lesser extent, in non-treated tau30 OHSC (Figure 5A; Figure S10). This shows that pS396 is triggered in tau30 slice cultures, especially when human BH (obtained from patients with AD as well as control subjects) is added to the culture medium, independently of subsequent tau pathology potentiation. Nevertheless, the co-localization of MC1+ cells with pS396+, with high intensity being found only in AD-seeded slices, strongly suggests that strong accumulation of pS396+ only occurs in a pathological context. Similar results were obtained for pS202/pT205/pS208, detected with the AT8 antibody, another tau PTM seen as abnormal but not necessarily pathological.^36^^,^^37^ AT8+ cells were indeed found in tau30 OHSC treated with both AD and control BH, and to a lesser extent, in non-treated tau30 OHSC, even if high AT8+ cells were only found in AD-seeded OHSC (Figure S11).

**Figure 5:**
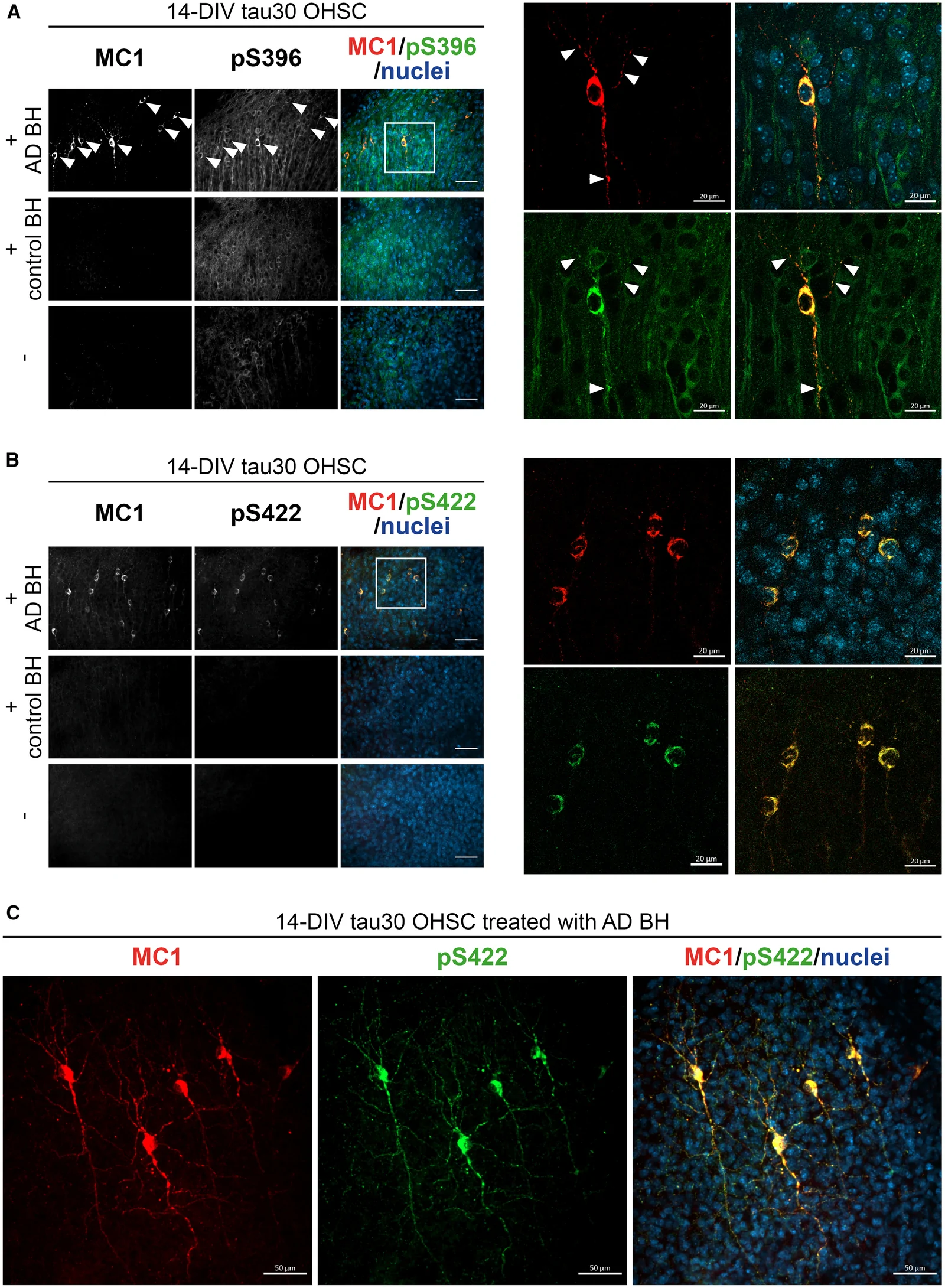
Characterization of the tau lesions observed in AD-seeded organotypic hippocampal slice cultures at 14 DIV (A and B) Representative images (A) of 14-DIV tau30 OHSC treated at 2 DIV with human BH obtained from an AD patient (top), a non-demented control subject (middle), or untreated (bottom), co-immunostained for MC1 (in red) and pS396 (in green). The nuclei were stained with DAPI (in blue). Higher magnification of the boxed area is shown on the right. Arrowheads indicate somas (left) and neurite-associated puncta (right) positive for both markers. Representative images (B) of 14-DIV tau30 OHSC treated at 2 DIV with human BH obtained from an AD patient (top), a non-demented control subject (middle), or untreated (bottom), co-immunostained for MC1 (in red) and pS422 (in green). Higher magnification of the boxed area is shown on the right. (A and B) The acquisitions (left) and the higher magnification acquisitions (right) were performed using a spinning disk microscope (20× objective) and a confocal microscope (63× objective), respectively. Scale bars, 50 μm (left) and 20 μm (right). (C) Representative image of 14-DIV tau30 OHSC treated at 2 DIV with human BH obtained from an AD patient co-immunostained for MC1 (in red) and pS422 (in green). The acquisitions were performed using a confocal microscope (40× objective). Scale bars, 50 μm.

The observation of highly intense MC1+ neuronal cell bodies at 14 DIV (Figures 3A and 5A) is in agreement with the report that in the brain of patients with AD, soma-mislocalized abnormal tau forms intracellular pre-tangles that then develop into mature NFT.^38^ pS422 is assumed to be a marker of pathological tau in pre-tangles and mature NFT.^39^ Consistently, the MC1+ somas observed in AD-seeded tau30 OHSC at 14 DIV were all found pS422+ and reciprocally (Figure 5B). pS422 staining was neither observed in tau30 OHSC treated with control BH nor in non-treated tau30 OHSC, as expected for a pre-tangle (and NFT) marker (Figure 5B). Thus, we unambiguously confirmed that only the treatment of tau30 OHSC with AD BH drives the development of MC1+ pS422+ pre-tangles.

Next, we wondered about the temporal relationship between early tau alterations and which event occurs first: namely, tau transconformation into conformers recognized by the MC1 antibody or pS422. Aside from the MC1+/pS422+ somas, MC1+/pS422+ puncta were massively observed along neurites in AD-seeded OHSC at 14 DIV (Figure 5C). At 7 DIV (meaning 5 DIV after treatment), MC1+ neurite-associated puncta were already present but fewer in number and less intense than at 14 DIV (Figure S12). Thus, we investigated the temporal relationship between MC1+ tau and pS422 at 7 DIV. We observed that the neurite-associated MC1+ puncta were rarely pS422+, while neurite-associated pS422+ puncta were very rarely observed, and the few ones were found to be MC1+ (Figure 6A). This strongly suggests that in AD-seeded OHSC, tau undergoes pathological (MC1+) transconformation before pathological pS422. Plasma pT217 being considered a specific and very early biomarker of AD^40^ prompted us to also test this marker even if recent data suggested that plasma pT217 is not strictly pathological.^41^ In AD-seeded OHSC, MC1+ puncta were only partially found pT217+ (Figure 6B), suggesting that tau also undergoes pathological transconformation before pathological pT217 in this model.

**Figure 6:**
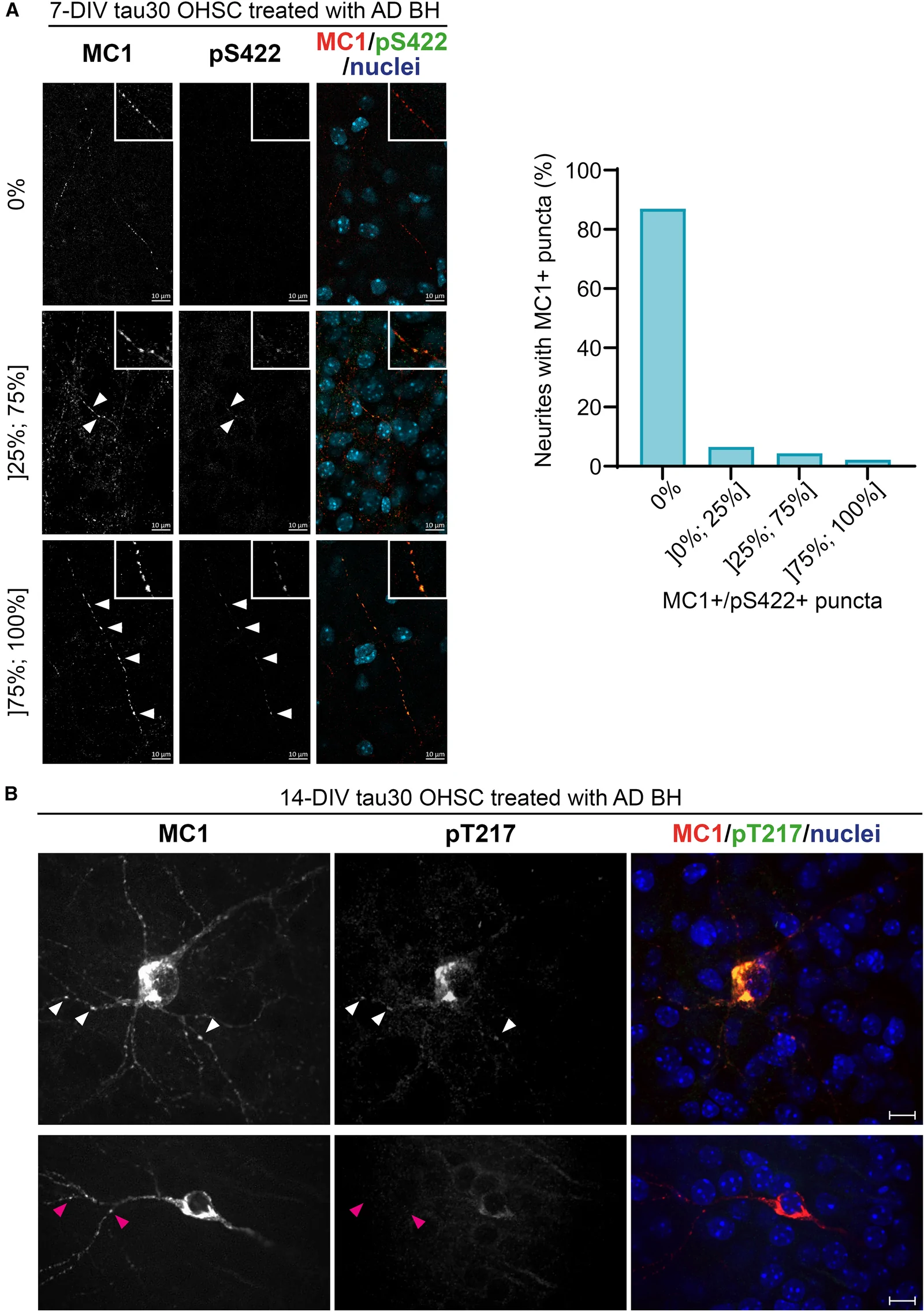
Characterization of the neurite-associated MC1+ puncta observed in AD-seeded organotypic hippocampal slice cultures (A) 46 neurites harboring MC1+ puncta in AD-seeded OHSC (from 2 independent OHSC experiments) at 7 DIV (treated at 2 DIV with BH from an AD patient) were classified into 4 groups according to the percentage of MC1+ puncta that were also pS422+: no MC1+ pS422+ puncta at all (0%), 25% or less of MC1+ pS422+ puncta (]0%; 25%]), between 25% and 75% of MC1+ pS422+ puncta (]25%; 75%]) and more than 75% of MC1+ pS422+ puncta (]75%; 100%]). On the left, images of 7-DIV tau30 OHSC treated at 2 DIV with human BH obtained from an AD patient co-immunostained for MC1 (in red) and pS422 (in green), as examples of the 0% group (top), the ]25%; 75%] group (middle) and the ]75%; 100%] group (bottom). The nuclei were stained with DAPI (in blue). The acquisitions were performed using a confocal microscope (63× objective). Arrowheads indicate puncta positive for both markers. Scale bars, 10 μm. At least 3 slices were analyzed per experiment. 21 and 25 neurites were observed in the first and second experiment, respectively. (B) Representative images of 14-DIV tau30 OHSC treated at 2 DIV with human BH obtained from AD patient co-immunostained for MC1 (in red) and pT217 (in green). The nuclei were stained with DAPI (in blue). The acquisitions were performed using a spinning disk microscope (63× objective). White and pink arrowheads indicate puncta positive for both markers and puncta only positive for MC1, respectively. Scale bars, 10 μm.

MC1+ puncta along neurites being the first detectable tau assemblies at 7 DIV (Figure 6A; Figure S12) would mean that, in AD-seeded OHSC, the seeding of tau is not initiated in the soma but in the neurites, even if later, pathological tau strongly accumulates in the soma. This hypothesis would mean that there are neurons with MC1+ staining in neurites, which is absent in the soma at that stage. To confirm this, we screened neurite-associated MC1+ puncta in AD-seeded OHSC co-labeled for MC1 and human tau (Tau Nter) in order to visualize the neuronal somas (Figure 7). By this approach, we observed that some neurons displayed neurite-associated MC1+ puncta but no MC1+ staining in the soma, supporting the involvement of neurites as the sites of the initial tau seeding process in AD-seeded OHSC.

**Figure 7:**
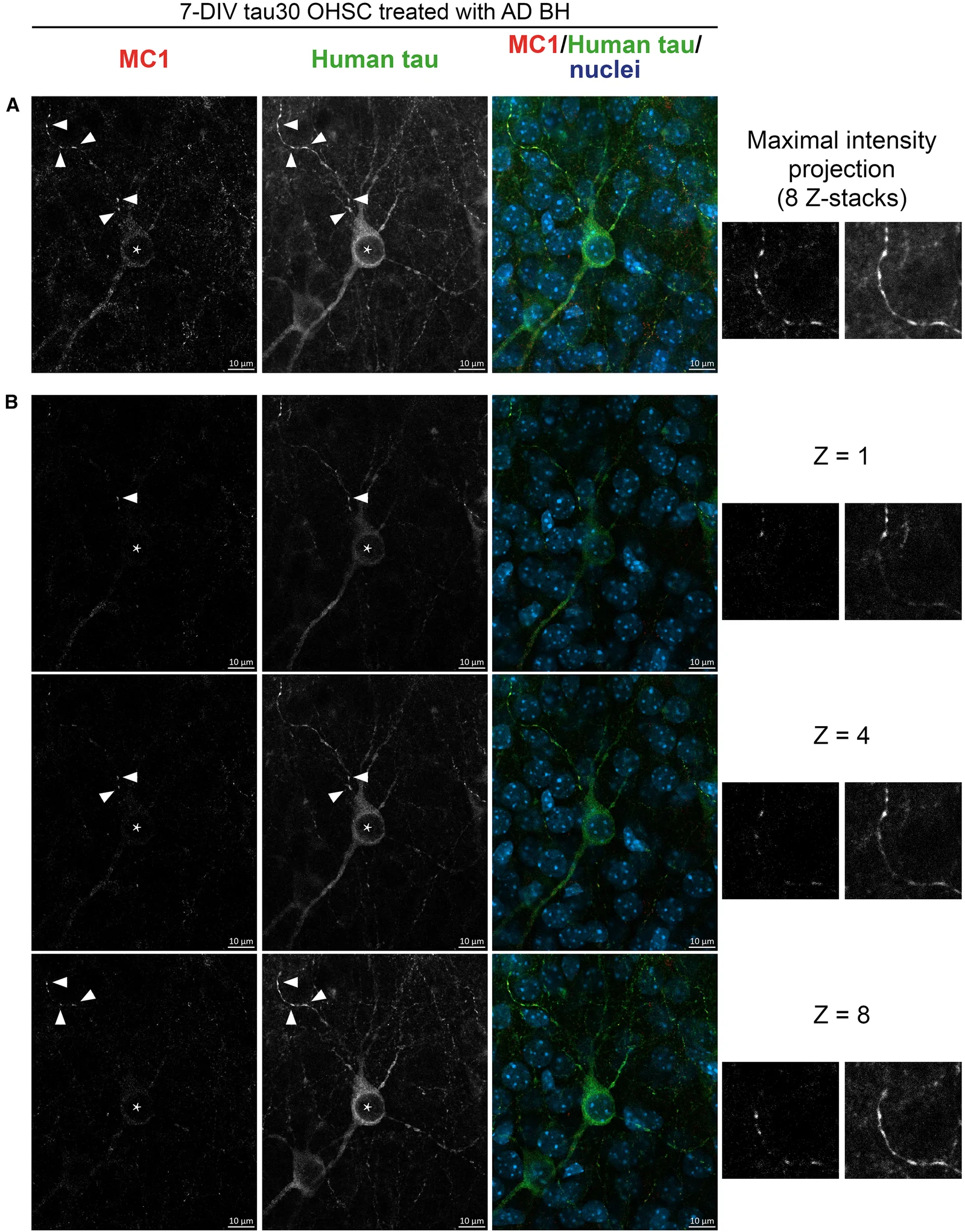
The neurite-associated MC1+ puncta appear first in AD-seeded organotypic hippocampal slice cultures Representative image of tau30 OHSC at 7 DIV, treated at 2 DIV with human BH obtained from an AD patient, co-immunostained for MC1 (in red) and human tau (rabbit polyclonal anti-tau Nter antibody, in green), showing a neuron with neurite-associated MC1+ puncta (indicated by arrowheads) and negative for MC1 in the soma (asterisk: nucleus). The nuclei were stained with DAPI (in blue). The acquisition was performed using a confocal microscope (63× objective), and a projection (maximal intensity) made of 8 single optical sections (A) and single optical sections (B) are presented, as well as details (right). Scale bars, 10 μm.

## Discussion

The present work shows the establishment of an *ex vivo* model, namely, of AD-seeded tau30 OHSC, which enables the study of tau pathology development in a highly accelerated manner. Indeed, pre-tangle structures can already be observed as early as 12 days after the application of AD BH. To our knowledge, this is the first demonstration that human tau seeds from AD brains can potentiate tau pathology in OHSC. Our data confirm that tau seeds are instrumental in this process, although other factors enriched in AD BH, such as Aβ, may also contribute.

Our in-depth analysis of this *ex vivo* potentiation revealed both misfolded and hyper- and abnormally- phosphorylated tau species. We further demonstrated *de novo* generation of seeding activity in AD-seeded tau30 OHSC using the FRET-based biosensor cell assay. However, we observed variability in measurements of tau seeding activity both in slice homogenates and in human BH. This variability may be partially attributed to differences between biosensor cell aliquots (derived from the same initial ATCC vial but amplified in our lab before storage in liquid nitrogen) and/or to potential modifications in seed content over one year, despite storage at −80°C in working aliquots to avoid freeze-thaw cycles (the two assays performed on the slice homogenates were conducted 14 months apart). Notably, variability between replicates within the same assay can only be attributed to the inherent variability of our *ex vivo* model, representing a limitation. Therefore, this model is better suited for deciphering the molecular and cellular mechanisms underlying tau seeding process and for testing therapeutic strategies, rather than for comparing tau seeding activity across a broad range of human tissues.

The AD-seeded tau30 OHSC model enabled us to revisit the chronology of tau pathology development *ex vivo*. We observed that pathological tau (MC1+) transconformation first occurs along neurites and only subsequently appears in neuronal cell bodies, consistent with previous observations in primary cell culture.^22^ These findings suggest that pathological tau initially forms in neurites before spreading to cell bodies, although this hypothesis requires formal demonstration. If confirmed, future studies should characterize the tau species involved in this process, including their quaternary structures and PTM. In the human AD brain, seeded aggregation is assumed to occur initially in axons rather than in somas, as tau is predominantly axonal. This assumption is based on the hypothesis that tau abundance is critical for seeding initiation. However, in our model, human tau is more widely expressed at the sub-cellular level, with high somatic expression in CA1 hippocampal neurons (Figure S6). The observation that tau misfolding follows a temporal sequence in neurites suggests that the initial seeding site is not determined by tau abundance alone. Rather, the restriction of pathological tau assemblies to neurites in AD-seeded OHSC strongly suggests that neurites possess a specific vulnerability that predisposes them to be the initial site of tau seeding, rather than the soma. One hypothesis is that misfolded tau accumulates in neurites due to their lower (though not absent) degradative capacity.^42^^,^^43^ This possibility warrants further investigation.

A major advantage of organotypic brain slice cultures is the ability to investigate how tau pathology affects neural networks and, conversely, how the network architecture and the cellular diversity influence tau pathology development, including the contributions of non-neuronal cell types such as microglia.^44^^,^^45^ While our initial observations did not reveal neuronal cell loss or microglial responses to tau pathology, more sensitive analyses (e.g., assessing synaptic loss, microglial and astrocytic morphologies) are clearly required. In a preliminary experiment, we investigated the neurite localization of pathological tau by co-immunostaining with pS422 and MAP2 (a dendritic marker; Figure S13). We observed predominantly axonal pathological tau, except in the immediate vicinity of pS422+ cell bodies, although more comprehensive studies are needed to confirm this finding.

Previous studies using organotypic brain slice cultures reported tau pathology-related neuronal death,^26^^,^^29^ but discrepancies with our findings may be explained by several factors. First, our study duration (14 DIV) may have been insufficient, and extending observations to three and four weeks might reveal cell death. Second, models based on overexpression of mutated tau RD^26^ or viral vector-induced overexpression of double/triple mutated human tau^29^ generated substantially more tau pathology over shorter time frames. Indeed, significant cell death may require very high levels of tau pathology. Supporting this, a previously published seeded-OHSC model with moderate tau pathology showed no tau pathology-related neuronal cell death even after four weeks.^30^ Importantly, no spontaneous tau pathology was observed in tau30 OHSC (even after one month of culture) or in P301S OHSC,^30^ unlike other organotypic brain slice culture studies. These differences may reflect variations in the type and/or expression level of human tau,^26^^,^^29^ as mutated tau RD is more aggregation-prone than other mutated tau forms, and the S320F mutation may be particularly pro-aggregative.^46^ Additionally, the brain regions used for the slice cultures and the presence of other mutated proteins (leading to increased Aβ accumulation) could influence spontaneous tau pathology development.^25^^,^^27^

Nevertheless, we detected pS396 and pS202/pT205/pS208 in control BH-treated OHSC, and to a lesser extent in untreated OHSC. The observation that our culture conditions, particularly the application of human control BH, induced these PTM without potentiating tau pathology aligns with the literature, as these modifications can occur in physiological contexts, including in young individuals without AD predisposition.^26^ Although these PTM have been shown previously to favor pathological tau (MC1+) transconformation,^7^ they are insufficient to drive tau aggregation in OHSC, at least within one month of culture. Additional factors, such as those discussed above, are likely required.

In contrast, pS422 was detected only in AD-seeded OHSC, linking this PTM strictly to pathology, consistent with its characterization as a pathological marker in the literature.^39^ Our data further suggest that, in the context of tau seeding, pS422 appears after pathological tau (MC1+) transconformation, potentially triggered or favored by the conformational change. However, this does not preclude pS422 from appearing first in spontaneous tau aggregation or from initiating transconformation and aggregation. While pS422 can be induced by temperature changes and may not be strictly pathological,^47^ detection methodology may also influence interpretation. Our immunostaining detects cells with pS422 tau levels above a certain threshold, whereas immunoblot could reveal collective increases in pS422 across cells, even if individual cells display low levels. Finally, our data strongly suggest that pT217 also appears after pathological (MC1+) transconformation in the context of tau seeding.

### Conclusion

This study presents the successful establishment of an AD-seeded tau30 OHSC model for investigating the mechanisms underlying tau transconformation, phosphorylation, and localization during tau pathology development, initially mediated by human-derived tau seeds. By faithfully mimicking tau pathology in an accelerated yet reliable manner within a brain-like environment, this model constitutes a highly valuable tool to bridge the gap between cellular and animal models.

### Limitations of the study

As discussed, we observed variability between slice cultures performed at different time points, limiting the model’s suitability for comparing tau seeding activity across a broad range of human tissues. Instead, it is better suited for deciphering mechanisms underlying tau seeding and testing therapeutic strategies. Additionally, while AD involves wild-type tau, our tau30 OHSC model overexpresses double-mutated tau. Nevertheless, the use of human mutated and overexpressed tau substantially accelerated tau pathology kinetics, representing a key advantage of this *ex vivo* model.

## Resource availability

### Lead contact

Requests for further information and resources should be directed to and will be fulfilled by the lead contact, Sophie Halliez (sophie.halliez@univ-lille.fr).

### Materials availability

This study did not generate new unique reagents.

### Data and code availability

• The datasets analyzed during the current study are available from the lead contact upon reasonable request.
• This paper does not report original code.
• Any additional information required to reproduce the experiments and/or reanalyze the data reported in this paper is available from the lead contact upon request.

## Acknowledgments

This work was performed with the assistance of the Plateformes Lilloises en Biologie et Santé (PLBS) - UAR 2014 - US 41, in particular the EOPS facility and the BioImaging Center Lille (BICeL). The authors thank David Blum (LilNCog) and the reviewers for their helpful comments that improved the manuscript. This work would not have been possible without the support of the tissue donors and their families. This work was partially funded by the Patients’ Association France Alzheimer et maladies apparentées (grant no FA_AAP2022-6253) and the Fondation I-SITE ULNE. R.T. was partially supported by a doctoral fellowship from the 10.13039/100014808Fondation Vaincre Alzheimer.

## Author contributions

S.H. designed and supervised this study with the help of M.C. and L.B. R.T. carried out the main experiments and main analysis. C.L., D.M., and C.F. participated in the establishment of the OHSC. M.O. and T.B. participated in the measurement of tau seeding activity via the FRET-based biosensor cell assay. M.O. helped with the tau immunodepletion experiments. S.E. helped with the preparation and the characterization of the brain homogenates. B.A. and C.-A.M. provided the human brain samples. R.T. and S.H. wrote the original draft. M.C., M.O., and L.B. edited the manuscript. All authors read and approved the final manuscript.

## Declaration of interests

The authors declare no competing interests.

## STAR★Methods

### Key resources table

**Table:** 

REAGENT or RESOURCE	SOURCE	IDENTIFIER
**Antibodies**		

Rabbit Anti-Tau Cter Antibody	Homemade (Tautou et al.^48^)	993-S1 [427-441]
Mouse IgG2a Anti-pTau (Ser422) Antibody, 2H9	Homemade (Troquier et al.^49^)	2H9
Mouse IgG1 Anti-pTau (Thr212, Ser214) Antibody, AT100	Thermo Fisher Scientific	Cat# MN1060, RRID:AB_223652
Mouse IgG1 Anti-Tau Nter Antibody, 7C12	Homemade (Guedjal et al.^50^)	7C12 [11-21]
Rabbit Anti-GAPDH Antibody	Sigma-Aldrich	Cat# G9545, RRID:AB_796208
Goat Anti-rabbit IgG Antibody, Peroxidase	Vector Laboratories	Cat# PI-1000-1, RRID:AB_2916034
Horse Anti-mouse IgG Antibody, Peroxidase	Vector Laboratories	Cat# PI-2000-1, RRID:AB_2336177
Mouse IgG1 MC1 Antibody	Gifted by Peter Davies (Albert Einstein College of Medicine; New York; USA)	Cat# MC1, RRID:AB_2314773
Rabbit Anti-pTau (Ser422) Antibody	Homemade (Bussière et al.^51^)	988
Rabbit Anti-pTau (Ser396) Antibody	Thermo Fisher Scientific	Cat# 44-752G, RRID:AB_2533745
Rabbit Anti-pTau (Thr217) Antibody	Thermo Fisher Scientific	Cat# 44-744, RRID:AB_2533741
Mouse IgG1 Anti-pTau (Ser202, Thr205) Antibody, AT8	Thermo Fisher Scientific	Cat# MN1020, RRID:AB_223647
Rabbit Anti-Human Tau Nter Antibody	Homemade: generated by immunization with a 12-21 tau peptide.	[12-21]
Mouse IgG1 Anti-MAP2 Antibody	Sigma-Aldrich	Cat# M4403, RRID:AB_477193
Rabbit Anti-Neurofilament 200 (NF200) Antibody	Sigma-Aldrich	Cat# N4142, RRID:AB_477272
Rabbit Anti-Glial fibrillary acidic protein (GFAP) Antibody	Dako	Cat# Z0334, RRID:AB_10013382
Rabbit Anti-Iba1 Antibody	Abcam	Cat# ab178846, RRID:AB_2636859
Mouse IgG1 Anti-NeuN Antibody	Sigma-Aldrich	Cat# MAB377, RRID:AB_2298772
Goat Anti-Mouse IgG1 Antibody, Alexa Fluor 568	Molecular Probes	Cat# A-21124, RRID:AB_141611
Goat Anti-Rabbit IgG Antibody, Alexa Fluor 488	Molecular Probes	Cat# A-11008, RRID:AB_143165
Goat Anti-Rabbit IgG Antibody, Alexa Fluor 568	Molecular Probes	Cat# A-11011, RRID:AB_143157

**Chemicals, peptides, and recombinant proteins**		

K18 fibrils	Gifted by Clément Danis and Isabelle Landrieu (CNRS EMR9002 - Integrative Structural Biology; Lille; France)	N/A

**Critical commercial assays**		

Nucleospin Tissue extraction kit	Macherey-Nagel	740952.250
Pierce^TM^ BCA Protein Assay kit	Thermo Scientific	23227
EZ-Link Sulfo-NHS-SS-Biotinylation kit	Thermo Scientific	21445
Streptavidin Mag Sepharose kit	Cytiva	28-9857-99

**Experimental models: Cell lines**		

Tau RD P301S FRET-based biosensor cells	ATCC	Cat# CRL-3275, RRID:CVCL_DA04
HEK 293T cells	ATCC	Cat# CRL-3216, RRID:CVCL_0063

**Experimental models: Organisms/strains**		

Tau30 hemizygous males	Breed on-site^16^	N/A
C57BL/6JRj females	Janvier Labs	RRID:IMSR_RJ:C57BL-6JRJ

**Oligonucleotides**		

Primers to genotype mouse pups	Published^52^	N/A

**Software and algorithms**		

ImageQuant TL software	Cytiva	v10.2
FACSDiva acquisition software	BD Biosciences	FACSDiva v7.0
Kaluza analysis software	Beckman Coulter	Kaluza v2
Zen Blue	Zeiss	v3.6 Blue edition
Fiji (ImageJ)	Fiji (ImageJ)	Fiji-win64
GraphPad Prism	GraphPad Software	GraphPad v10.2.0

**Other**		

Vibratome	Leica	VT1200S

### Experimental model and study participant details

#### Human brain samples

Human brain samples from non-demented controls (Ctl, *n* = 5) and patients with AD (*n* = 5) were obtained from the Lille Neurobank (fulfilling French legal requirements concerning biological resources and declared to the competent authority under the number DC-2008-642) with donor consent, data protection, and Ethics Committee approval. Samples were managed by the CRB/CIC1403 Biobank, BB-0033-00030. The demographic data are listed in Table 1.

#### Organotypic hippocampal slice cultures

The experiments were carried out with the approval of an ethics committee (agreement APAFIS#43474-2023050714441306 v6 from CEEA75, Lille, France) and follows European guidelines for the use of animals in research.

Tau30 hemizygous males^16^ and C57BL/6JRj females (Janvier Labs) were housed in a temperature-controlled room (20°C–22°C) and maintained on a 12-h day/12-h night cycle with food and water provided *ad libitum*. The brains were dissected from post-natal day (P) 7 tau30 hemizygous pups and wild-type (WT) littermates obtained from breeding the tau30 hemizygous males and C57BL/6JRj females. The genotype was assayed using polymerase chain reaction on DNA extracted from the pups’ tails using a kit according to the recommendations of the manufacturer (Nucleospin Tissue extraction kit, Macherey-Nagel). The dissection was performed in ice-cold Gey’s balanced salt solution (GBSS, G9779, Sigma), which was supplemented with the glutamate receptor antagonist kynurenic acid (1 mM; K3375, Sigma) and glucose (33 mM; 25-037-Cl, Corning). The brains were embedded in low melting point agarose (2% in supplemented GBSS; 1670, Euromedex) and a vibratome (VT1200S, Leica) was used to cut horizontally 350 *μ*m thick slices in ice-cold supplemented GBSS. Hippocampal slices were microdissected and collected under a binocular magnifying glass before being plated on cell culture inserts (PICM03050, Millipore) and cultured in 1 mL of slice culture medium (SCM) in 6-well plates (353046, Dutscher). SCM was composed of 50% Minimum Essential Medium Eagle with Hank's salt and sodium (M5775, Sigma), 25% Basal Medium Eagle (B1522, Sigma), and 25% heat-inactivated horse serum (26050-088, Gibco) and supplemented with 6% glucose, antibiotic-antimycotic (15240-096, Gibco), and L-glutamine (25030-624, Thermo Fisher). OHSC (4 to 6 slices per culture insert) were cultured at 37 °C in a humidified atmosphere containing 5% CO_2_ and the medium was changed every 2 days in a sterile class II biosafety cabinet (Thermo Scientific PSM HERASAFE KS).

### Method details

#### Preparation of human brain homogenates

Human brains samples were homogenized with a glass potter at 10% (w/v) in sterile phosphate-buffered saline (PBS) and briefly sonicated using a vibracell-72405 sonicator (BioBlock Scientific) in a sterile class II biosafety cabinet (Thermo Scientific).

#### Seeding of organotypic hippocampal slice cultures

Human BH obtained from AD patients (or non-demented controls) were briefly sonicated in a sonication bath (Diagenode) for 30 sec before being added into SCM of OHSC at two days *in vitro* (DIV). Unless otherwise stated, the human BH were used at a final concentration of 80 μg/ml total protein. After 16 h (overnight) the SCM was replaced with regular SCM and OHSC were cultured as described above.

#### Protein sample quantification

The total protein concentration of the samples was determined using a BiCinchoninic acid Assay (BCA) kit according to the recommendations of the manufacturer (Pierce^TM^ BCA Protein Assay kit, ThermoScientific). After incubation of the samples with the reagent for 30 min at 37 °C, the absorbance was read at 570 nm (Multiskan FC, ThermoScientific).

#### Western blot analysis

Human BH were diluted with lithium dodecyl sulfate 2X supplemented with reducing agents (Invitrogen). Unless otherwise stated, 15 μg of total protein were separated on 4–12% Criterion Bis–Tris Gels (Bio-Rad) then proteins were blotted onto a nitrocellulose membrane (Cytiva, Amersham Protran, 0.45 μm). The membrane was blocked with 5% non-fat dry milk (for Tau Cter, GAPDH and Tau Nter 7C12) or 5% bovine serum albumin (BSA, NZYtech) (for 2H9) in TNT buffer solution (Tris 15 mM pH8, NaCl 140 mM, 0.05% Tween) or not blocked (for AT100). Next, membranes were incubated with primary antibodies overnight at 4 °C and then incubated with the corresponding secondary antibodies: peroxidase labeled horse anti-rabbit and/or anti-mouse for 45 min at room temperature (RT). Signals were revealed using a chemiluminescence assay (ECL, RPN2209, Amersham Bioscience) and visualized using a LAS 4000 imaging system (GE Healthcare). Results were quantified using ImageQuant software to allow normalization.

#### Propidium iodide staining

To assess cell viability, OHSC were incubated with 10 μg/mL of propidium iodide (PI, P3566, Molecular Probes) in SCM for 1 h at 37 °C before acquiring images under a fluorescence microscope (Leica MC120 HD).

#### Immunofluorescence analysis

After 7 or 14 DIV, OHSC were fixed in 4% paraformaldehyde (PFA) for 30 min at 4 °C then permeabilized and saturated with 0.1% Triton X-100 (Sigma) and 1% BSA (NZYtech) in PBS. Slices were incubated with primary antibodies overnight at 4 °C and then with the corresponding secondary antibodies for 2 h at RT. Nuclei were stained with 4′,6-diamidino-2-phenylindole (DAPI, 62248, Fisher Scientific) for 30 min at RT. Then slices were mounted on a slide and covered by a coverslip with fluoromount-G (00-4958-02, Invitrogen). Wide field acquisitions were performed using a fluorescent microscope (Zeiss Axio Imager Z1). For quantifications of the number of MC1-positive cell bodies, acquisitions were performed using a spinning disk microscope (20X objective, Yokogawa CSU-X1 spinning disk confocal on a Zeiss Axio Observer Z1) and counting was performed from Z-stacks using the “events” module of the Zen blue software (v3.6 Blue edition, Zeiss). Single optical sections from Z-stacks are displayed on the figures except if stated otherwise. Acquisitions of MC1 and phospho-tau/human tau/MAP2 co-labeling were performed using a confocal microscope (40X/63X oil objective, LSM 980, Zeiss) or a spinning disk microscope (20X/40X/63X oil objective) and individual Z-stack slices are displayed on the figures except if stated otherwise.

#### Measurement of tau seeding activity by FRET-based biosensor cell assay

Tau RD P301S FRET-based biosensor cells (CRL-3275, ATCC) and HEK 293T cells (CRL-3216, ATCC, used as negative controls for flow cytometry analysis) were plated into a 12-well plate (150,000 cells per well; 353043, Dutscher) and cultured in Dulbecco’s modified Eagle’s medium without HEPES (13345-364, Gibco) complemented with 10% fetal bovine serum (A52567-01, Gibco), 1% penicillin/streptomycin (15140-122, Gibco) and 1% GlutaMAX (35050-061, Gibco) for 24 h at 37 °C in a humidified atmosphere containing 5% CO2. Tau seeding activity was estimated of human BH (40 μg of total protein in 100 μL) and of 14-DIV OHSC homogenates (each replicate encompasses two slices collected in 100μL of PBS and then sonicated in a sonication bath for 30 sec). Sonicated K18 fibrils diluted in Opti-MEM (31985-070, Gibco) were used as positive controls and PBS was used as negative control. The transfection mixture (100 μL of each sample to analyze or control + 10 μL of Lipofectamine 2000 (11668019, Invitrogen) + 90 μL of Opti-MEM) was incubated for 20 min at RT and added to the cells. After 72 h, the cells were collected in warm PBS, and cell death was evaluated by adding Zombie NIR for 30 min at RT (1:200; Zombie NIR fixable viability kit, 423105, BioLegend). After one rinse in PBS, cells were fixed in 2% PFA for 10 min at RT and finally suspended in PBS for flow cytometry analyses using the flow cytometer Aria SORP (BD Biosciences) for the human BH or the flow cytometer SP6800 Spectral Analyser (Sony Corporation) for the OHSC homogenates and the FACSDiva acquisition software (v7.0, BD Biosciences) with the following excitation/emission wavelengths: excitation 405 nm, cyan fluorescent protein (CFP) emission 466 ± 40 nm and FRET yellow fluorescent protein (YFP) 529 ± 30 nm; excitation 488 nm, YFP emission 529 ± 30 nm. The FRET data were quantified using the Kaluza analysis software (v2, Beckman Coulter). Results are expressed as the percentage of FRET-positive cells × MFI (median fluorescence intensity).

#### Immunodepletion

The tau antibody (tau Nter 7C12, Homemade)^50^ was first biotinylated using the EZ-Link Sulfo-NHS-SS-Biotinylation Kit (Thermo Scientific) according to the recommendations of the manufacturer. Immunodepletion of tau from BH was performed using magnetic beads (streptavidin Mag Sepharose Kit, Cytiva, 28985799), according to the manufacturer’s instructions with minor modifications. 50 mL of Mag Sepharose beads were incubated with 5 mg of tau antibody or no antibody for 30 min with rotation at RT. After washing with 500 mL of washing buffer, the beads-antibody complex and the beads alone were incubated with 100 mg/mL of total protein of AD BH diluted in 300 μL of binding buffer for 60 min with rotation at RT. The beads-antibody-antigen complex and the beads alone were isolated using a magnetic holder, and the supernatants were collected as the tau immunodepleted fraction (tau-ID) and the control fraction (bead-ID), respectively. Beads-bound tau was then eluted using the elution buffer and stored as the immunocaptured tau fraction (tau-IC). Corresponding control was bead-IC. The total protein concentrations of the ID fractions were determined by BCA and all the fractions were analyzed by western blots. The fractions were stored at 4 °C before being used to treat the OHSC as previously described.

#### Iba1 immunofluorescence quantification

Iba1 immunolabeling was performed on OHSC at 14 DIV. Fluorescence images were acquired using a spinning-disk confocal microscope with a 20× objective. All images were acquired using identical acquisition parameters and controlled with Zen Blue software. Image analysis was performed using Fiji (ImageJ, Fiji-win64). For each hippocampal slice, two representative fields of view were acquired, and four slices were analyzed per experimental condition. A threshold was applied identically to all images to ensure consistency across experimental groups. Iba1 immunoreactivity was quantified by measuring the percentage of Iba1+ area within each field of view.

### Quantification and statistical analysis

Statistical analyses were carried out using Prism 10 software (GraphPad, v10.2.0). Comparisons of two groups were done using a Mann-Whitney U test. Comparisons of three or more independent groups were done using a Kruskal–Wallis test followed by a Dunn’s multiple comparisons test. Differences were considered significant when *p* < 0.05. Correlation analyses were done using Spearman’s rank correlation coefficient (r_s_) for the data obtained from the 10 human BH. For visual representation of the correlations, the raw data were plotted with simple linear regression lines and corresponding confidence intervals (dotted lines). These lines were included for visualization only, as Spearman's rank correlation assesses monotonic relationships, which are not necessarily linear.
