# Supplementary material for: Deciphering early tau pathology events using an Alzheimer’s disease-seeded organotypic hippocampal slice culture model
Source: iScience. 2026 Nov 2;29(11):118083. doi: 10.1016/j.isci.2026.118083 (PMC13755596; doi:10.1016/j.isci.2026.118083)
Supplement: Supplementary file 1 [file mmc1.pdf]

iScience, Volume *29*

Supplemental information

Deciphering early tau pathology events using an Alzheimer's disease-seeded organotypic hippocampal slice culture model

Rislane Taouili, Camille Lefebvre, Marie Oosterlynck, Thomas Bouillet, Sabiha Eddarkaoui, Dylan Musiol, Coraille Formentin, Bertrand Accart, Claude-Alain Maurage, Morvane Colin, Luc Buée, and Sophie Halliez

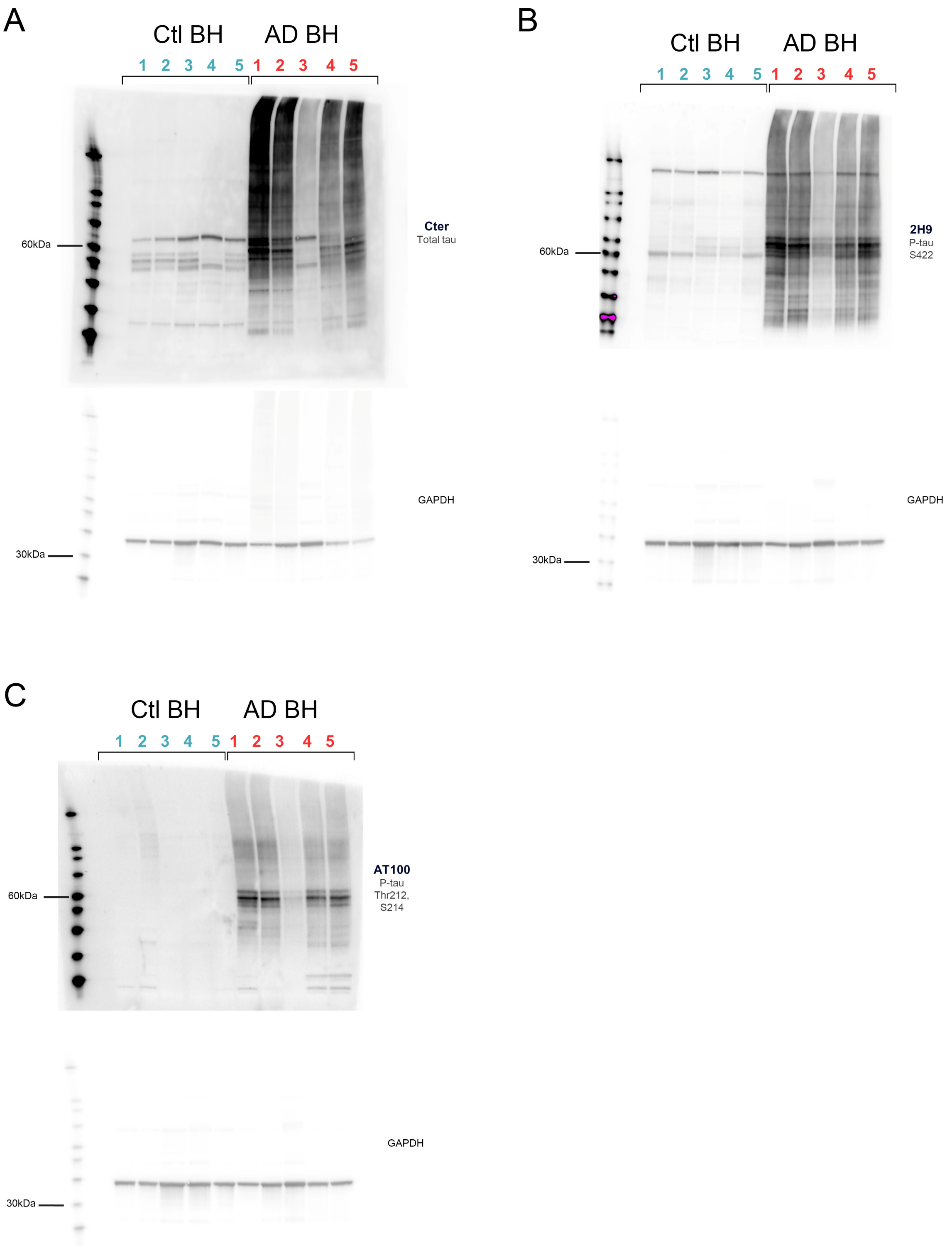

Figure S1. Uncropped images of the immunoblots displayed in Figure 1A.

Uncropped western blots using Cter (A), 2H9 (B) and AT100 (C) antibodies. GAPDH was used as a loading control.

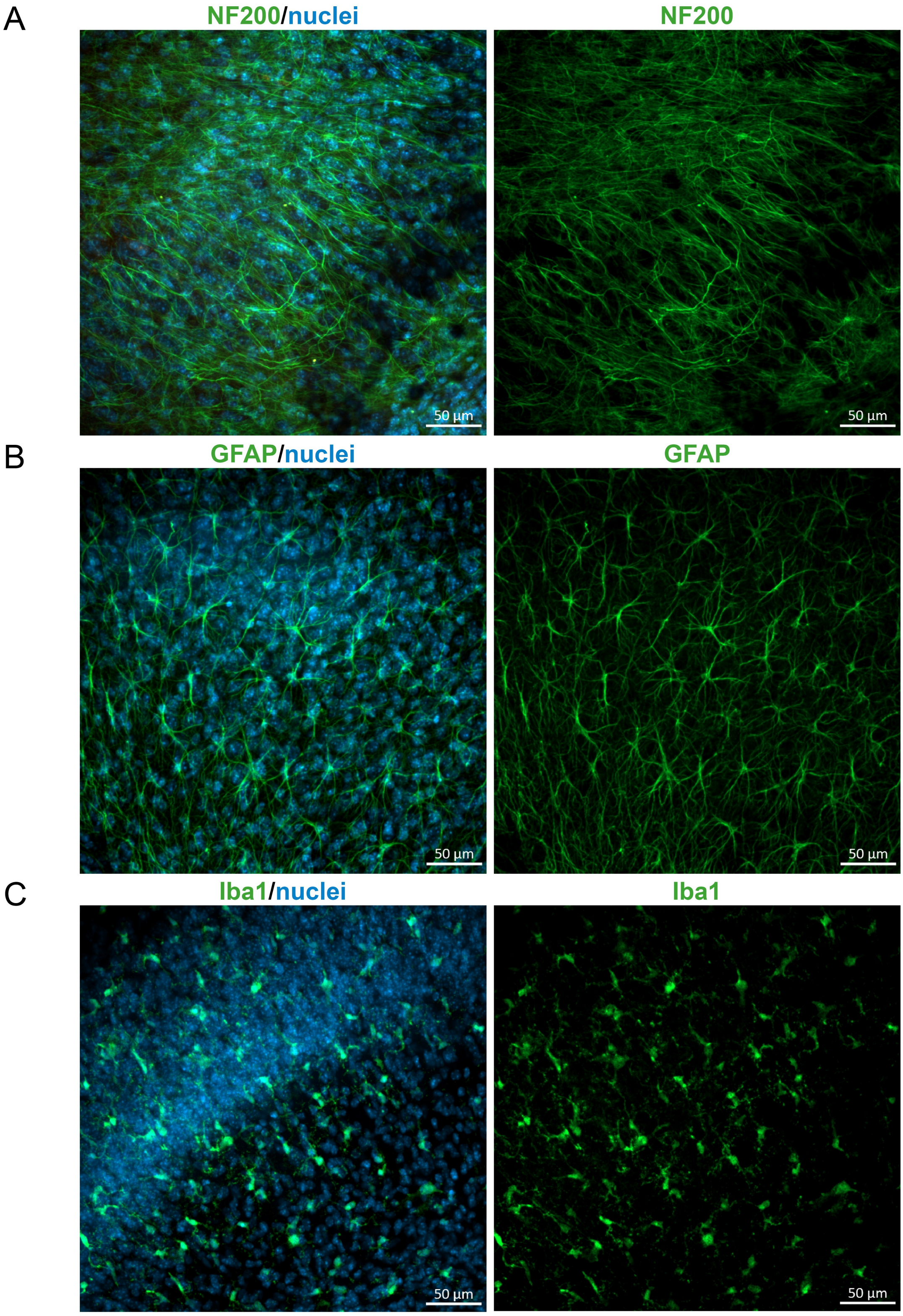

Figure S2. Tau30 organotypic hippocampal slice cultures maintain cellular diversity.

Representative images of tau30 OHSC at 14 DIV. Neuronal cells/neurofilaments were stained with NF200 antibody (A, in green), astrocytes were stained with GFAP antibody (B, in green) and microglial cells were stained

with Iba1 antibody (C, in green). The nuclei were stained with DAPI (in blue). Projections (maximal intensity) are presented. The acquisitions were performed using a spinning disk microscope (20X objective). Scale bars = 50 µm.

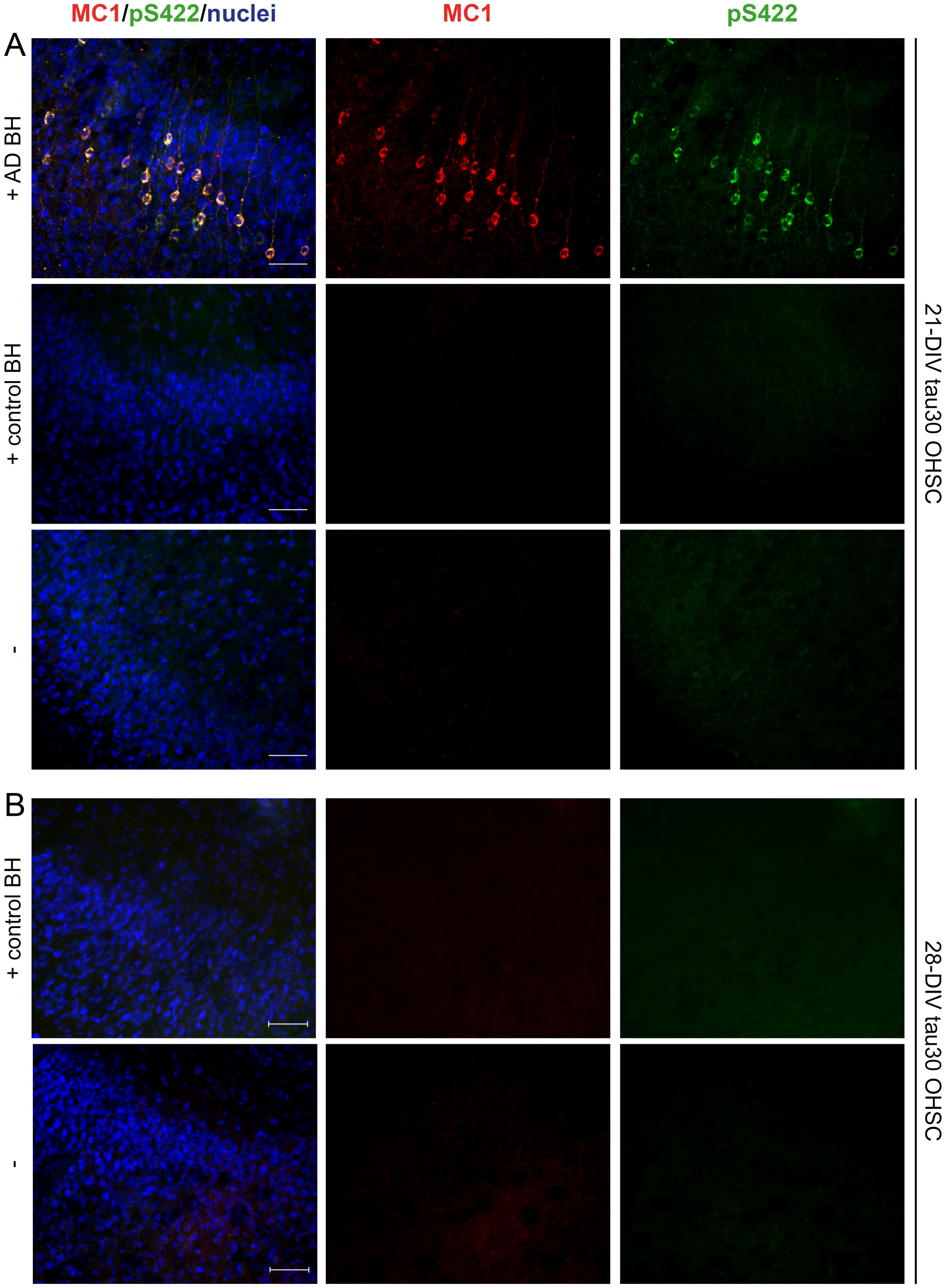

Figure S3. Tau pathology does not develop in control BH-treated or non-treated tau30 organotypic hippocampal slice cultures over four weeks.

Representative images of (A) 21-DIV tau30 OHSC treated at 2 DIV with human BH obtained from AD patient (top) or non-demented control subject (middle) or non-treated (bottom) and (B) 28-DIV tau30 OHSC treated at 2 DIV with human BH obtained from non-demented control subject (top) or non-treated (bottom). All slices were co-immunostained for MC1 (in red) and pS422 (in green). The nuclei were stained with DAPI (in blue). The acquisitions were performed using a spinning disk microscope (20X objective). Scale bars = 50 µm.

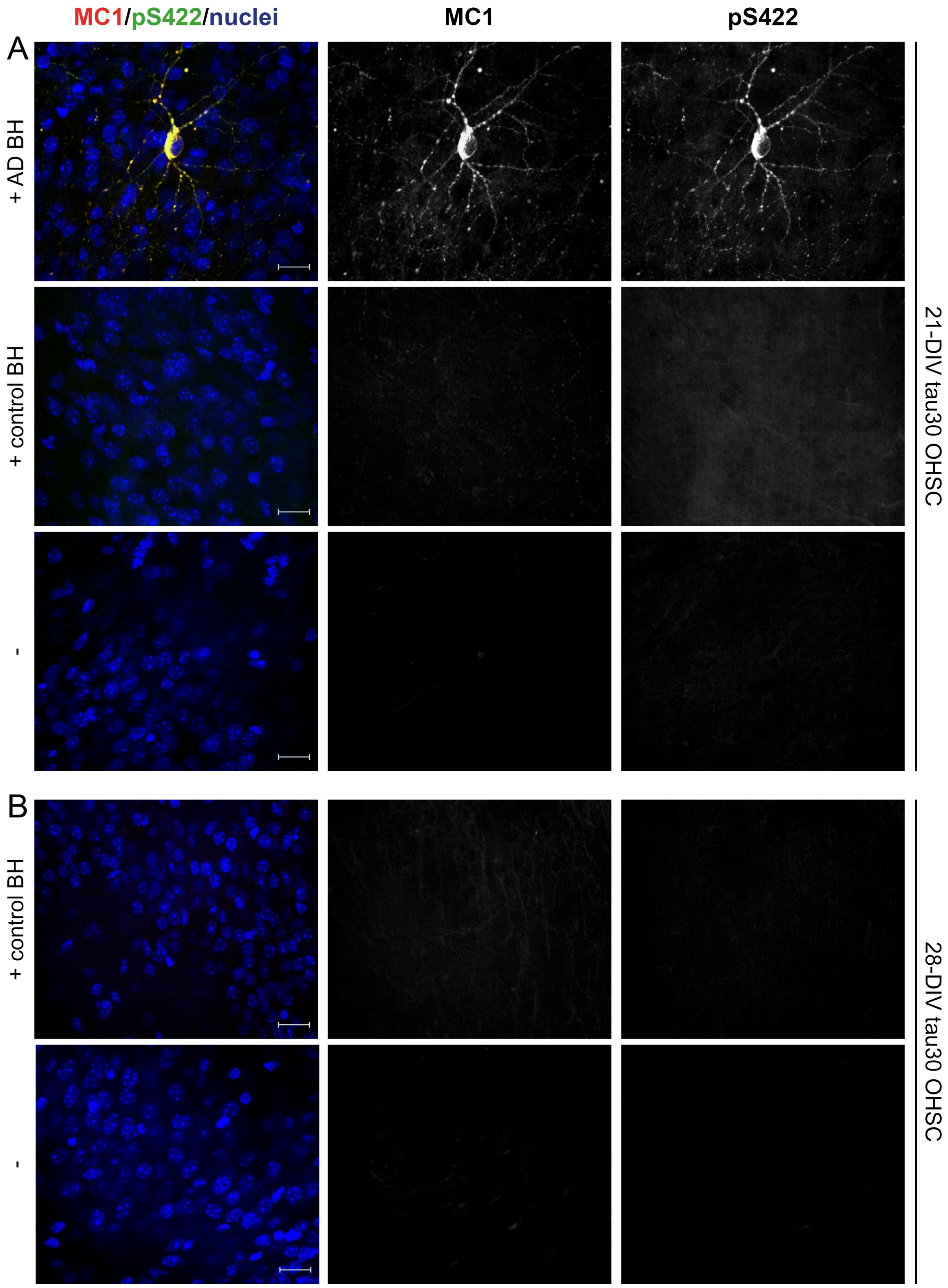

Figure S4. Neuronal bodies with tau pathology were not found in control BH-treated or non-treated tau30 organotypic hippocampal slice cultures.

Representative images of (A) 21-DIV tau30 OHSC treated at 2 DIV with human BH obtained from AD patient (top) or non-demented control subject (middle) or non-treated (bottom) and (B) 28-DIV tau30 OHSC treated at 2 DIV with human BH obtained from non-demented control subject (top) or non-treated (bottom). All slices were co-immunostained for MC1 (in red) and pS422 (in green). The nuclei were stained with DAPI (in blue). The acquisitions were performed using a spinning disk microscope (40X objective). Scale bars = 20 µm.

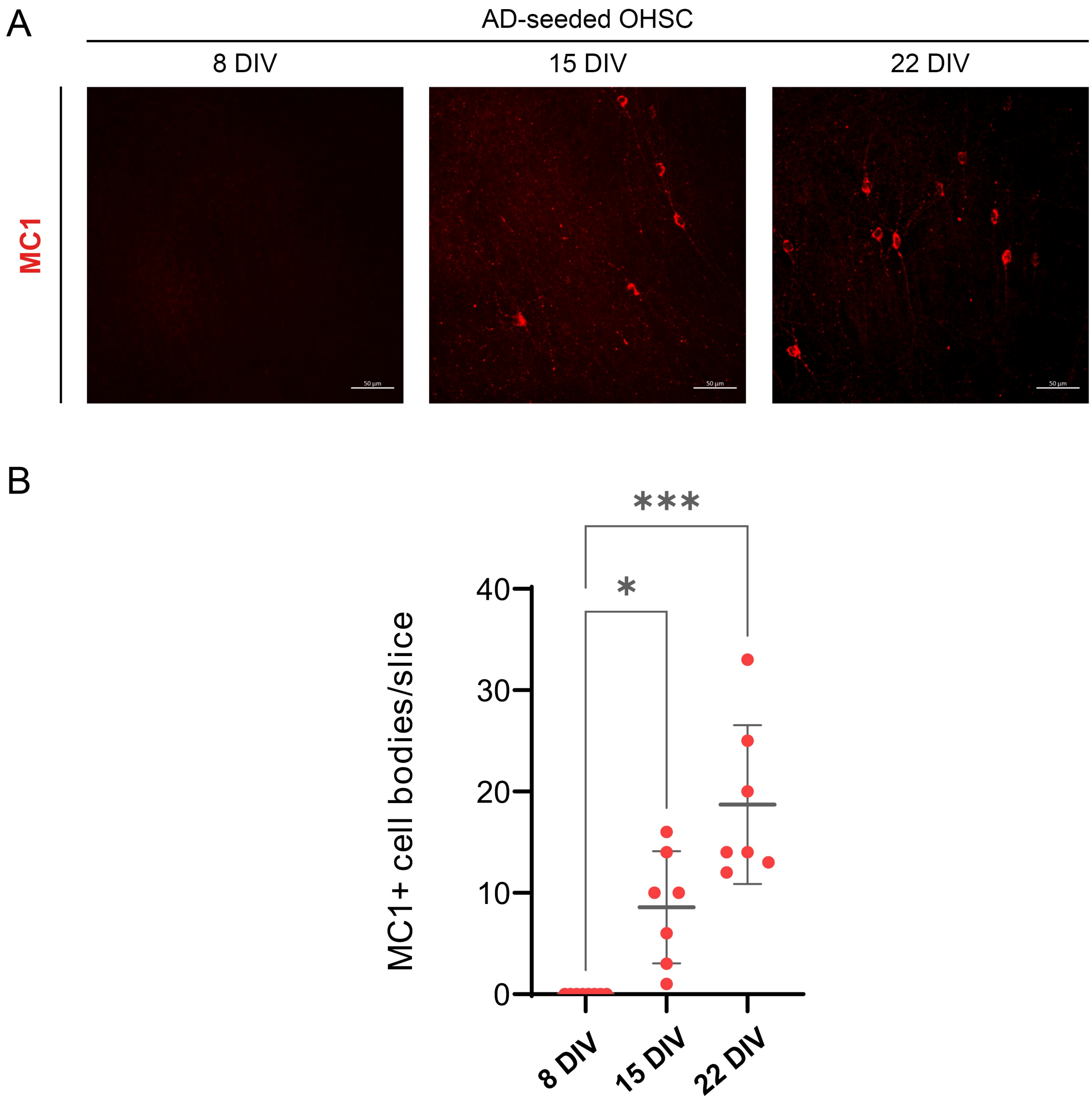

Figure S5. Tau pathology potentiation increases over time in AD-seeded OHSC.

(A) representative images of MC1 labeling (in red) in tau30 OHSC at 8 (left), 15 (middle) or 22 (right) DIV, all previously treated at 2 DIV with human BH obtained from an AD patient. The acquisitions were performed using a spinning disk microscope (20X objective). Scale bars = 50 µm.

(B) number of MC1+ cell bodies per tau30 OHSC at 8, 15 and 22 DIV, all previously treated at 2 DIV with human BH obtained from an AD patient. *: $p < 0.05$, ***: $p < 0.0001$, Dunn's multiple comparisons test.

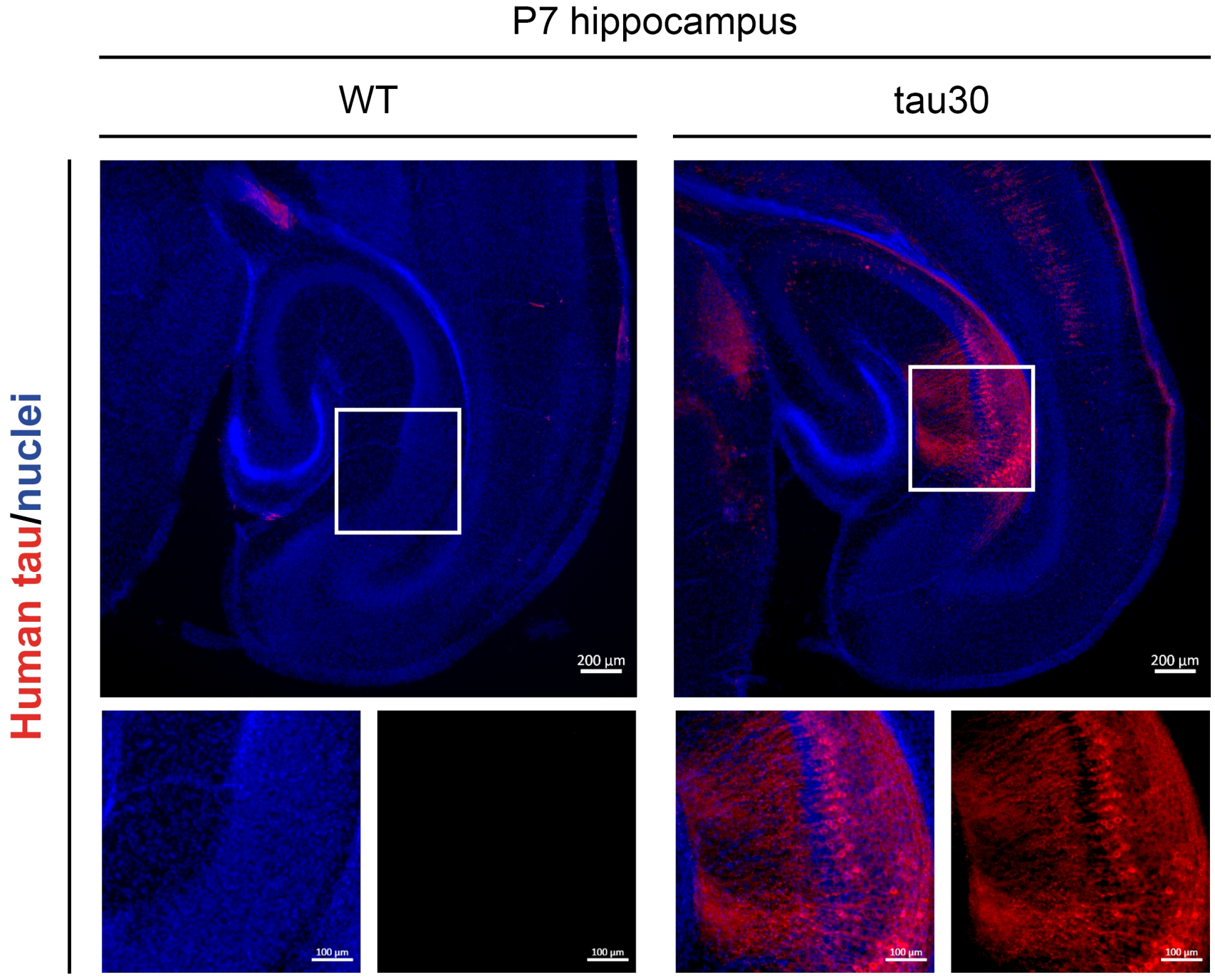

Figure S6. Expression pattern of human tau protein in the hippocampus of tau30 P7 mice.

Immunostaining for human tau was performed on hippocampal sections from WT (left) and tau30 (right) 7-day-old mice. Human tau was labeled with the rabbit polyclonal anti-tau Nter antibody (in red) and the nuclei were labeled with DAPI (in blue). The wide field acquisitions (top) and the higher magnifications acquisitions focused on the CA1 sub-region (bottom) were performed using a fluorescent microscope (5X and 20X objectives, respectively). Scale bars = 200 µm (top) and 100 µm (bottom).

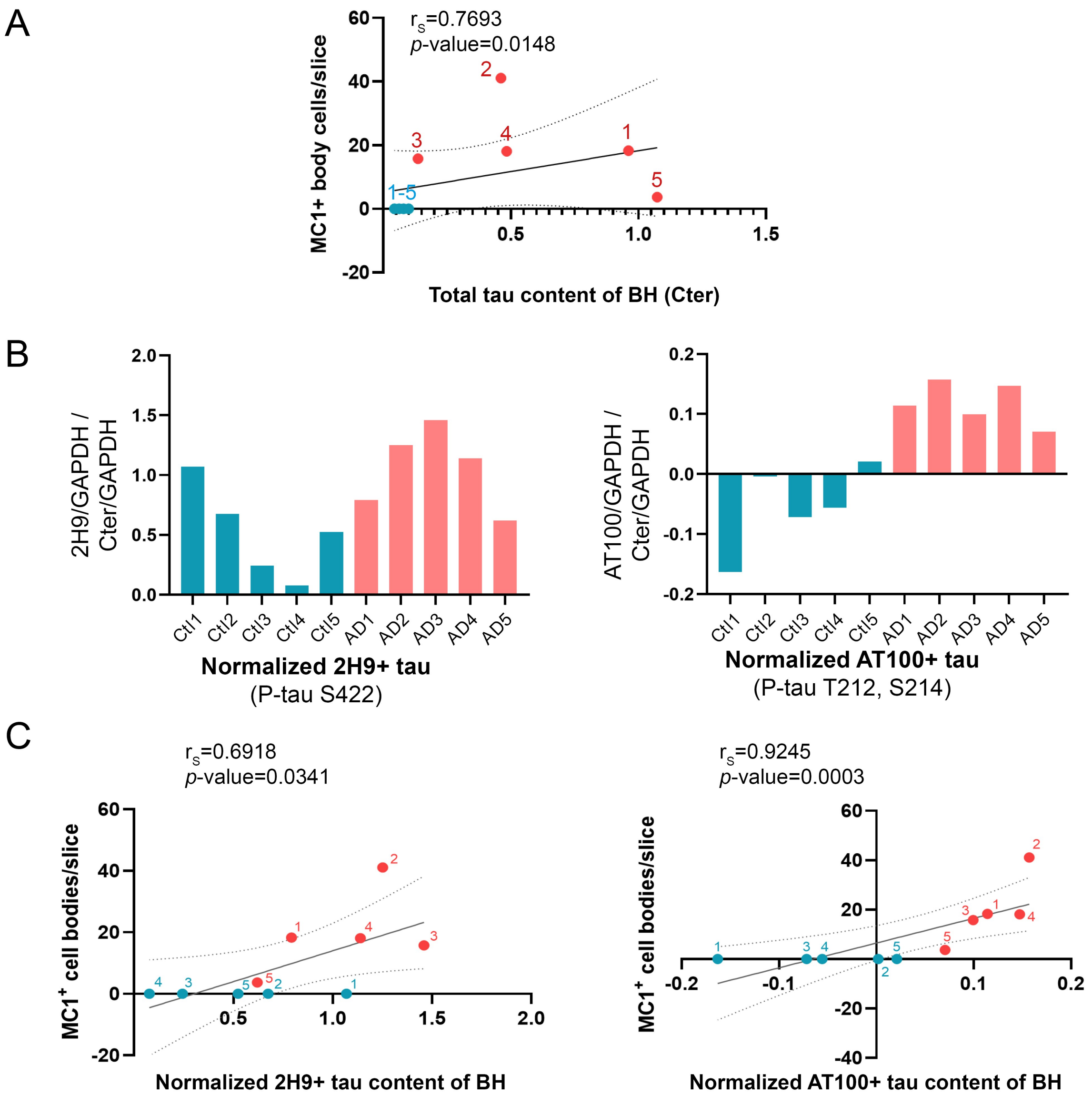

Figure S7. Additional correlations between the level of tau pathology potentiation and the tau content of the human brain homogenates.

(A) Mean number of MC1+ cell bodies per BH-treated tau30 OHSC obtained for each human BH (AD and control BH represented in red and blue, respectively) plotted as a function of the total tau content of the BH quantified from the immunoblots performed with tau Cter and anti-GAPDH antibodies presented in Figure 1. r_s and *p*-value are reported on the graph. The linear regression line and confidence interval were added under the assumption of a linear relationship.

(B) Relative quantifications of phosphorylated tau at residue Ser422 (2H9, left) and phosphorylated tau at both residues Thr212 and Ser214 (AT100, right) normalized to the total tau content of the human BH.

(C) Mean number of MC1+ cell bodies per BH-treated tau30 OHSC obtained for each human BH (AD and control BH represented in red and blue, respectively) plotted as a function of the normalized pathological tau content of the BH presented in (B). r_s and *p*-values are reported on the graphs. The linear regression lines and respective confidence intervals were added under the assumption of a linear relationship.

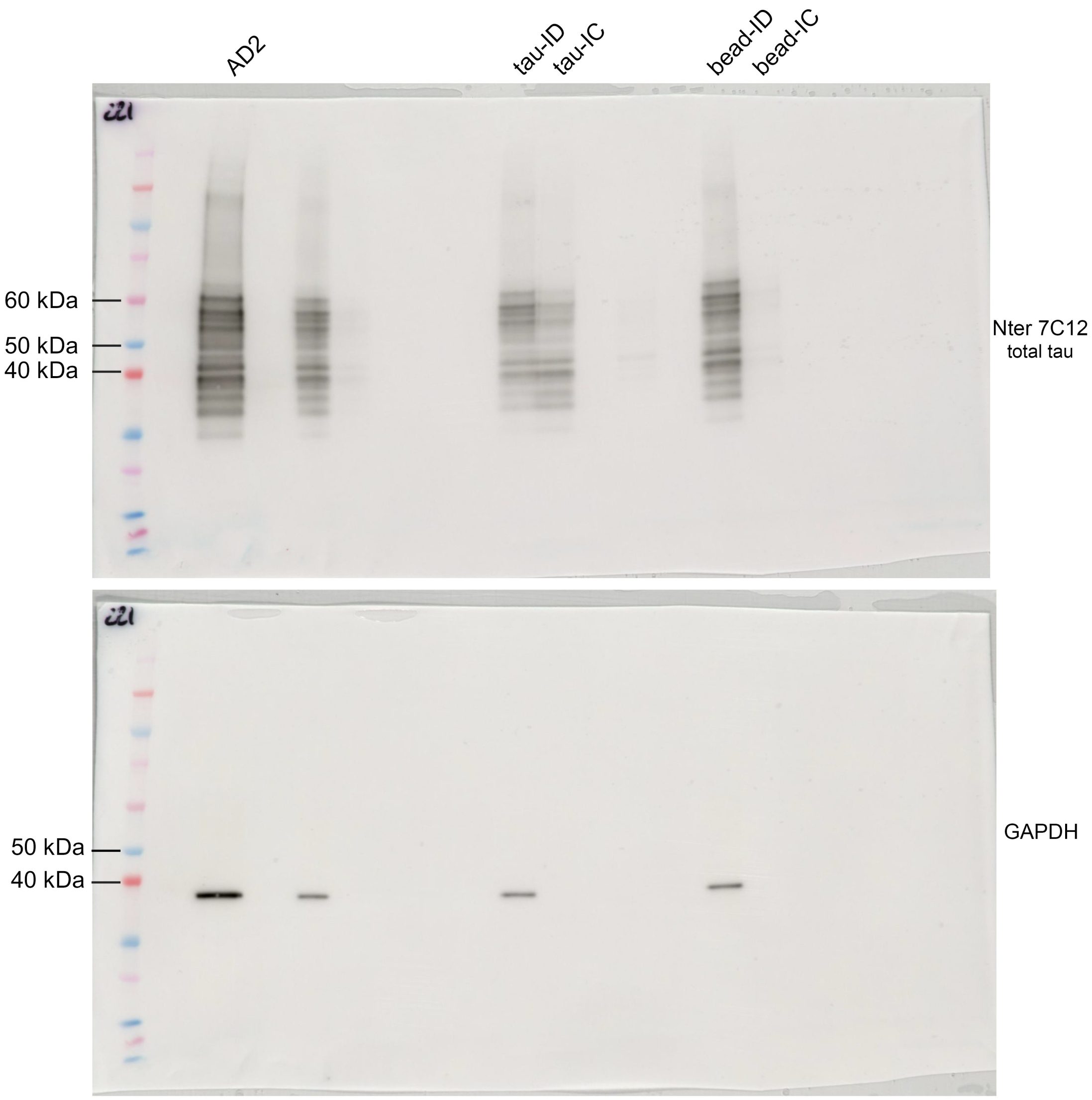

Figure S8. Uncropped image of the western blot displayed in Figure 3E.

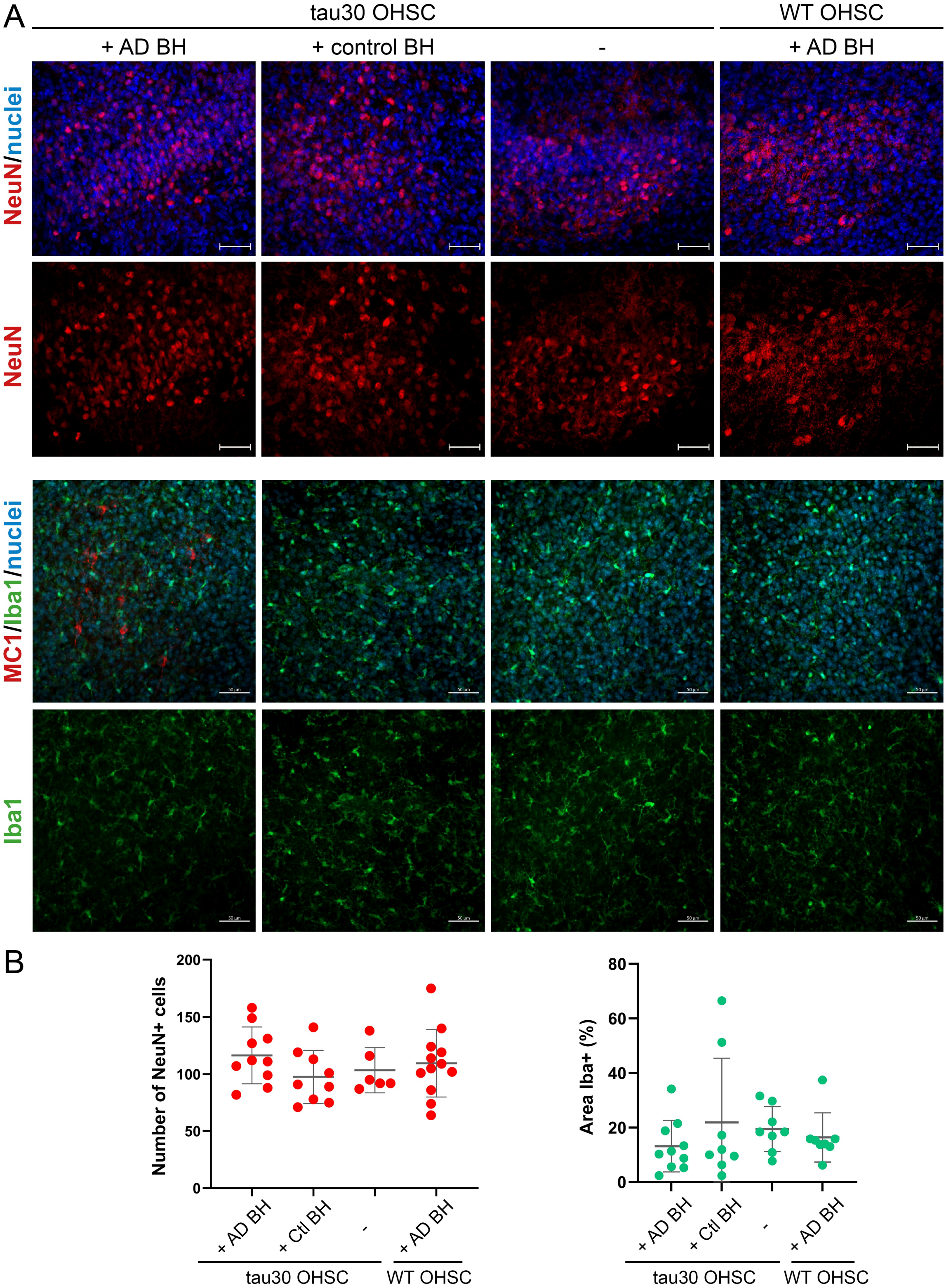

Figure S9. Neither neuronal loss nor microglial response were observed in AD-seeded organotypic hippocampal slice cultures compared to controls at 14 DIV.

(A) Representative images of tau30 OHSC at 14 DIV previously treated at 2 DIV with human BH obtained from AD patient or non-demented control subject or non-treated and WT OHSC at 14 DIV previously treated at 2 DIV with human BH obtained from AD patient (from left to right). OHSC were immunostained for NeuN (in red, top) or MC1 and Iba1 (in red and green, respectively, bottom). The nuclei were stained with DAPI (in blue). The acquisitions were performed using a spinning disk microscope (20X objective) and for NeuN immunostainings, projections (maximal intensity, 2 µm) are presented. Scale bars = 50 µm.

(B) Number of NeuN+ cells (left) and percentage of Iba1+ area (right) within each field of view for tau30 OHSC at 14 DIV previously treated at 2 DIV with human BH obtained from AD patient or non-demented control subject or non-treated and WT OHSC at 14 DIV previously treated at 2 DIV with human BH obtained from AD patient.

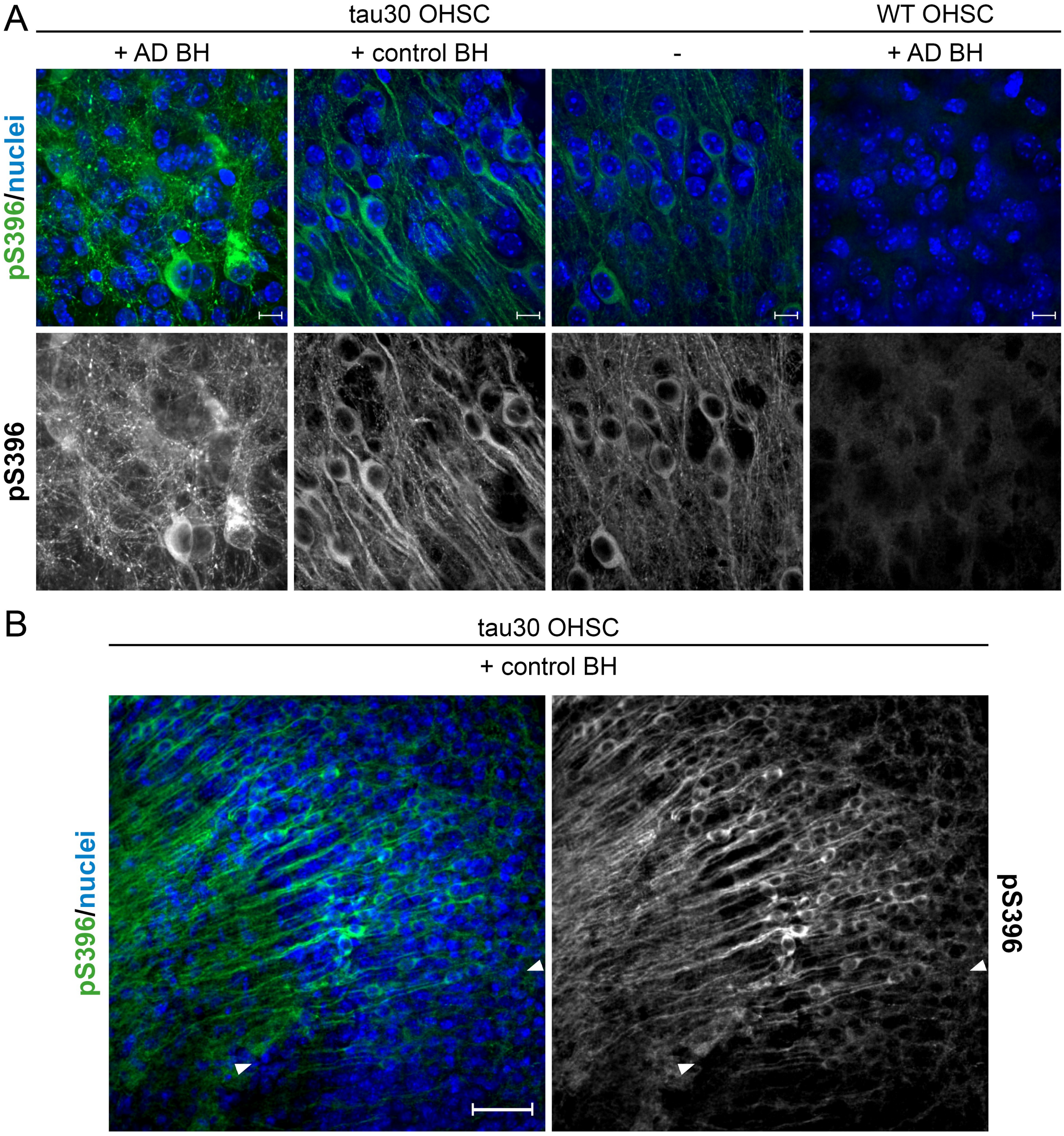

Figure S10. Abnormal tau phosphorylation (pS396) in organotypic hippocampal slice cultures.

(A) Representative images of tau30 OHSC at 14 DIV previously treated at 2 DIV with human BH obtained from AD patient or non-demented control subject or non-treated and WT OHSC at 14 DIV previously treated at 2 DIV with human BH obtained from AD patient (from left to right) immunostained for pS396 (in green). The nuclei were stained with DAPI (in blue). The acquisitions were performed using a spinning disk microscope (63X objective). Scale bars = 10 µm.

(B) Representative images of tau30 OHSC at 14 DIV previously treated at 2 DIV with human BH obtained from non-demented control subject immunostained for pS396 (in green). The nuclei were stained with DAPI (in blue).

Arrowheads indicate the border between pS396+ and pS396- neurons that corresponds to the CA1/CA2 border. The acquisition was performed using a spinning disk microscope (20X objective). Scale bar = 50 µm.

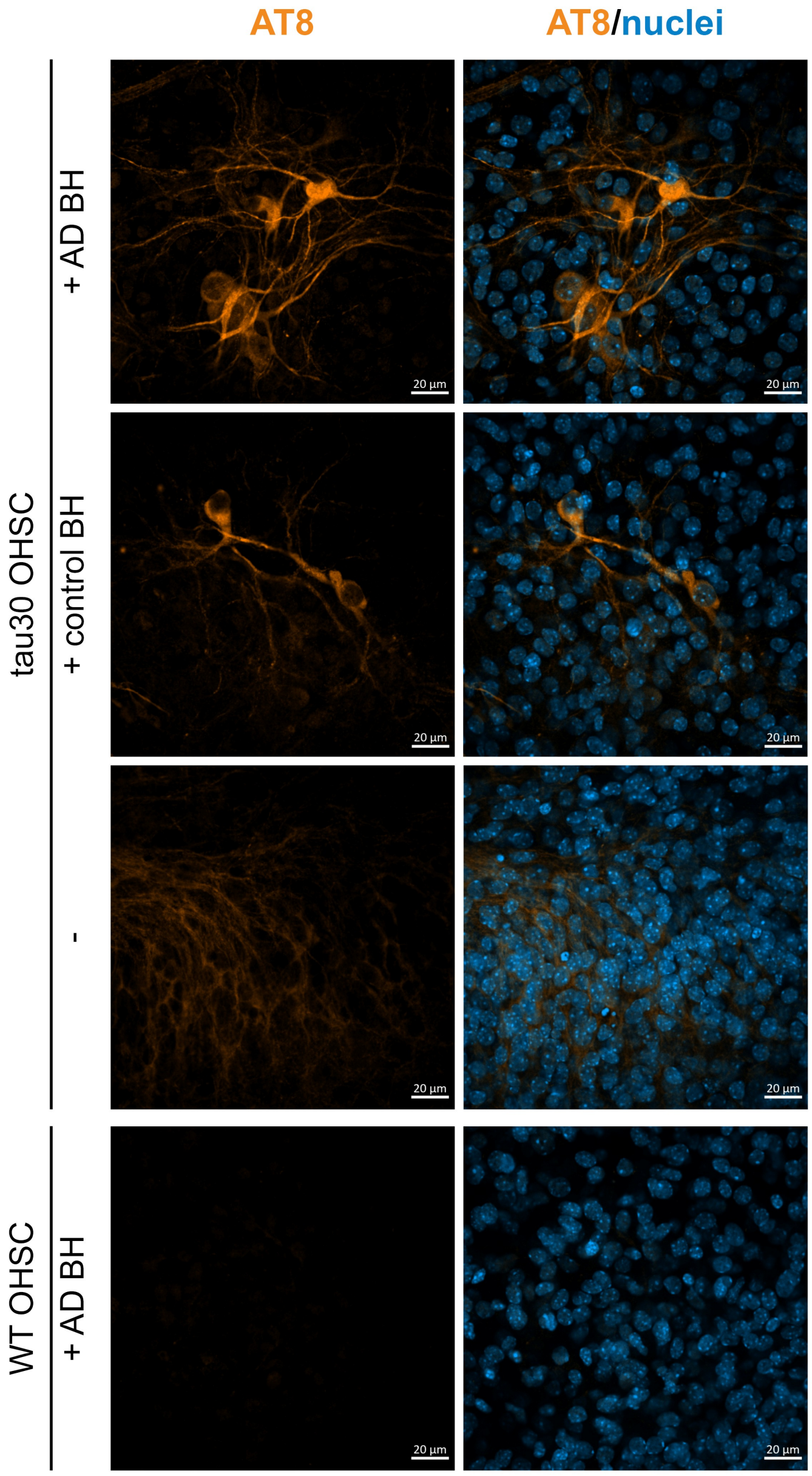

Figure S11. Abnormal tau phosphorylation (AT8) in organotypic hippocampal slice cultures.

Representative images of tau30 OHSC at 14 DIV previously treated at 2 DIV with human BH obtained from AD patient or non-demented control subject or non-treated and WT OHSC at 14 DIV previously treated at 2 DIV with human BH obtained from AD patient (from top to bottom). pS202/pT205/pS208 was stained with AT8 antibody (in orange) and the nuclei were stained with DAPI (in blue). The acquisitions were performed using a spinning disk microscope (40X objective). Scale bars = 20 µm.

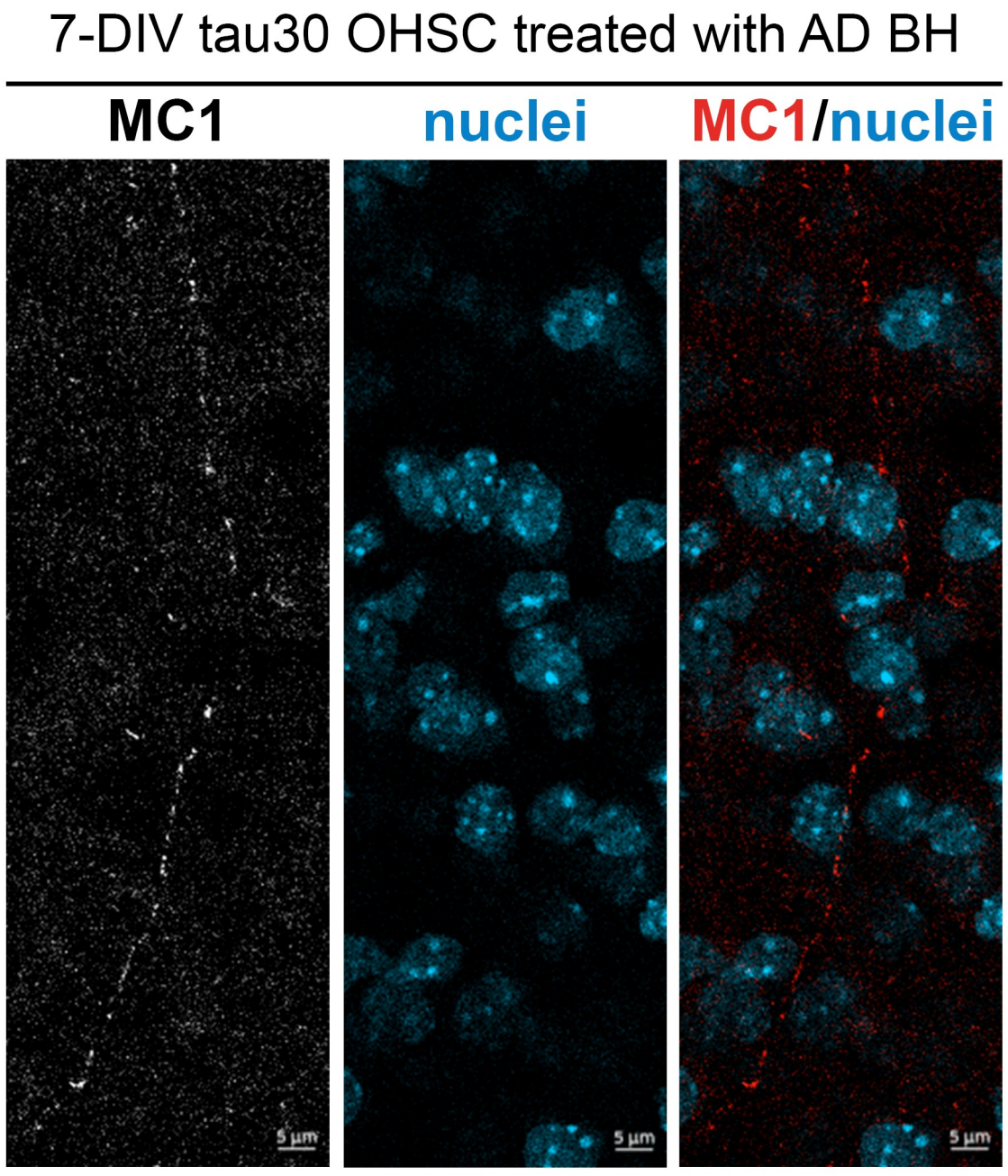

Figure S12. Neurite-associated MC1+ puncta observed in AD-seeded organotypic hippocampal slice cultures at 7 DIV.

Representative image of 7-DIV tau30 OHSC treated at 2 DIV with human BH obtained from AD patient immunostained for MC1 (in red). The nuclei were stained with DAPI (in blue). The acquisition was performed using a spinning disk microscope (63X objective). Scale bars = 5 µm.

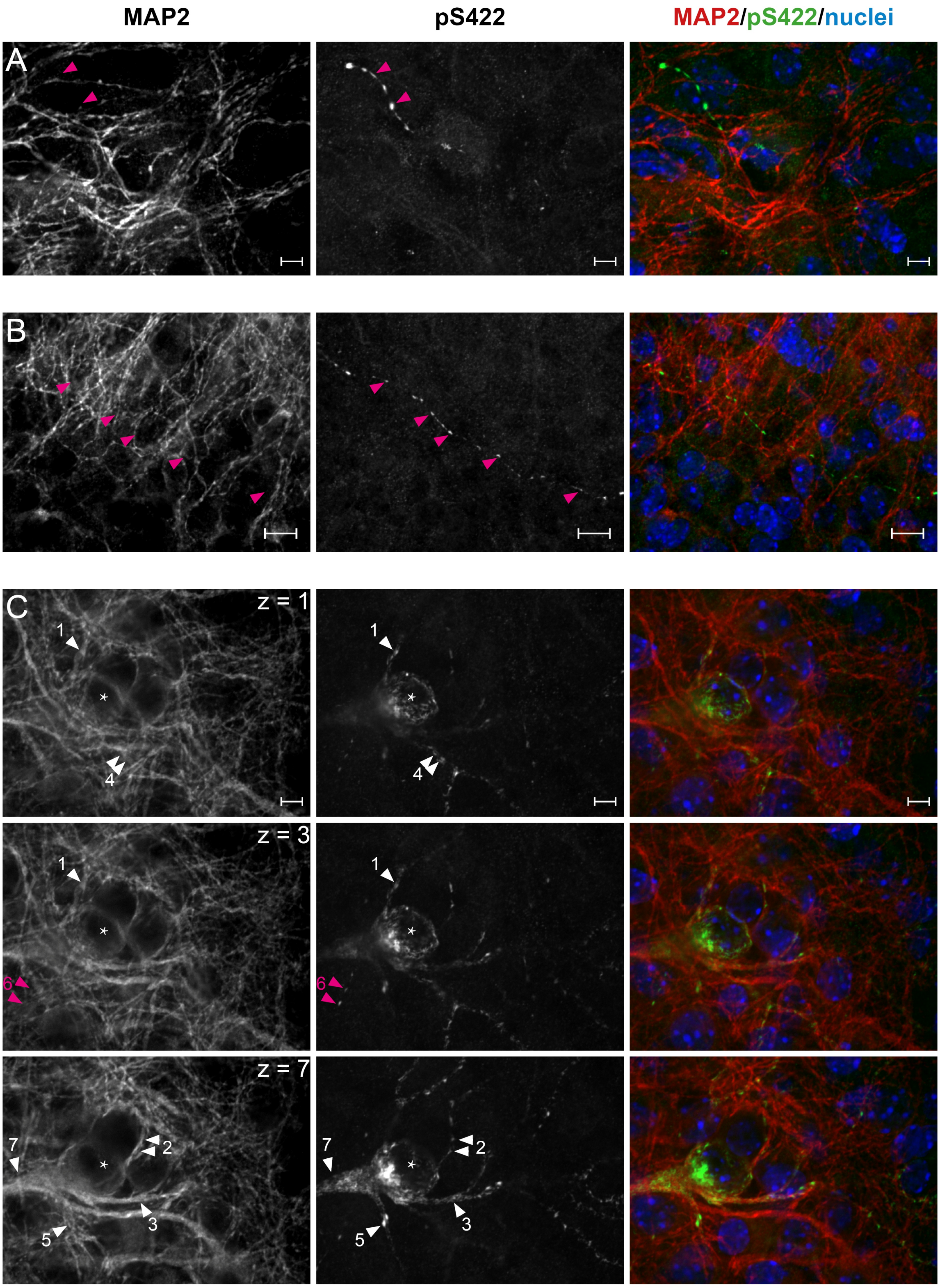

Figure S13. Neurite-associated pS422+ puncta observed in AD-seeded organotypic hippocampal slice are mainly present along axons except at proximity of pS422+ cell bodies. (A-C) Representative image of 14-DIV tau30 OHSC treated at 2 DIV with human BH obtained from AD patient immunostained for MAP2 (in red) and

pS422 (in green). The nuclei were stained with DAPI (in blue). Pink arrowheads indicate pS422+ neurites that are negative for MAP2 staining (axons) while white arrowheads indicate pS422+ neurites that are positive for MAP2 staining (dendrites). Asterisk indicates a pS422+ cell body surrounded by seven neurites. The acquisitions were performed using a spinning disk microscope (A, C, 100X objective and B, 63X objective). (A, C) scale bars = 5 µm. (B) scale bars = 10 µm.
